# Regiodefined synthesis of brominated hydroxyanthraquinones related to proisocrinins

**DOI:** 10.3762/bjoc.12.52

**Published:** 2016-03-16

**Authors:** Joyeeta Roy, Tanushree Mal, Supriti Jana, Dipakranjan Mal

**Affiliations:** 1 Department of Chemistry, Indian Institute of Technology, Kharagpur- 721302, India, Fax: +913222282252

**Keywords:** brominated anthraquinones, Darzens condensation, Hauser annulation, proisocrinins

## Abstract

Dibromobenzoisofuranone **12**, synthesized in six steps, was regiospecifically annulated with 5-substituted cyclohexenones **13**/**36** in the presence of LiO*t*-Bu to give brominated anthraquinones **14**/**38** in good yields. Darzens condensation of **30** was shown to give chain-elongated anthraquinone **32**. Alkaline hydrolysis of **38** furnished **39** representing desulfoproisocrinin F.

## Introduction

**Figure 1:**
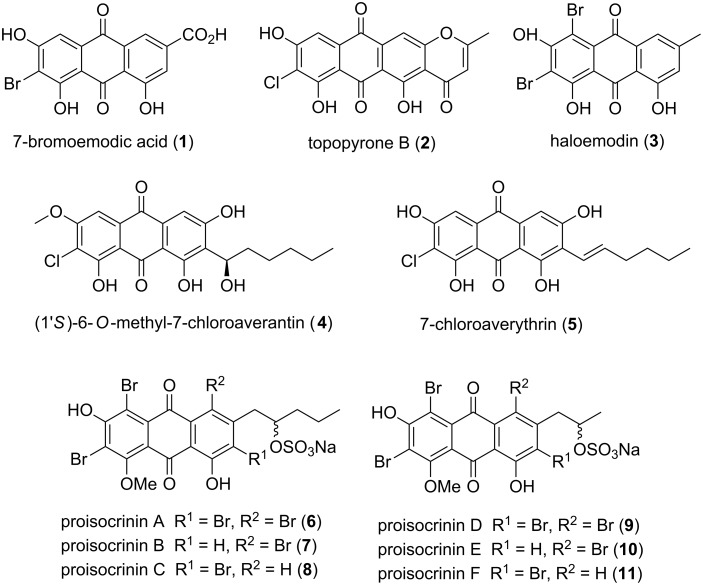
Halogenated anthraquinones.

Anthraquinones constitute the largest group of naturally occurring quinones [1–5]. Isolated mainly from fungal sources, they display a wide range of biological activities which include anti-inflammatory, antifungal, antiparasidal, and cytotoxic properties [6–11]. Anthraquinones are well-known as colorants in foods, drugs, and textile industries. They are also used as chemical sensors and liquid crystals [1–5]. Halogenated anthraquinones form a minor group of natural pigments [12–15]. 7-Bromoemodic acid (**1**), isolated from the crinoid *Holopus rangii*, shows remarkable cytotoxic activities. Topopyrone B (**2**) stabilizes DNA topoisomerase I and DNA topoisomerase II. Haloemodin (**3**) acts as an antibacterial agent inhibiting DNA gyrase and bacterial topoisomerase I. 6-*O*-Methyl-7-chloroaveratin (**4**) displays potent inhibitory activity against human tumor cell lines SF-268, MCF-7, and NCI-H460, with IC_50_ values of 7.11, 6.64, and 7.42 μM, respectively [12]. Proisocrinins A–F (**6**–**11**), recently isolated from the stalked crinoid *Proisocrinus ruberrimus* (Figure 1) are the first water soluble natural anthraquinone pigments, and show promising antifeedant properties [16].

A brief literature survey revealed that the routes for the synthesis of anthraquinones are primarily based upon five categories, such as Friedel–Crafts reactions, Hauser annulations, Diels–Alder reactions, transition metal-mediated reactions and biomimetic aldol condensations [17–23], and reports on the synthesis of brominated anthraquinones are scare [12–15]. Having inspired by the convergence and the regiochemical integrity of the Hauser annulation [24–30], we explored it for the construction of the bromoanthraquinone scaffolds of proisocrinins **6**–**11**.

## Results and Discussion

### First synthetic route

**Scheme 1:**
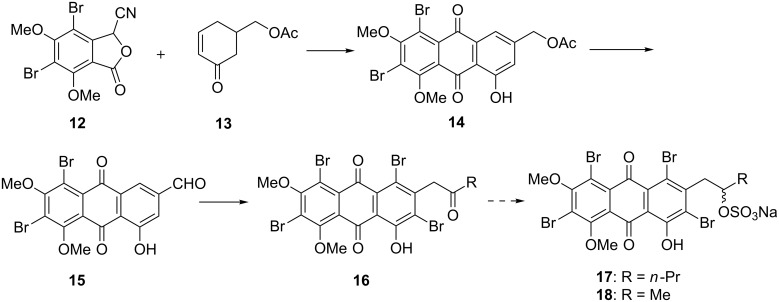
Initially proposed synthetic scheme for proisocrinins **6**–**11**.

Anthraquinone **14** was proposed to be synthesized by the Hauser annulation of cyanophthalide **12** and cyclohexenone **13** (Scheme 1). A functional group manipulation of **14** was expected to give anthraquinone carboxyaldehyde **15**. Employment of a Darzens condensation followed by bromination was considered for further elaboration of **15** to **16**.

**Scheme 2:**
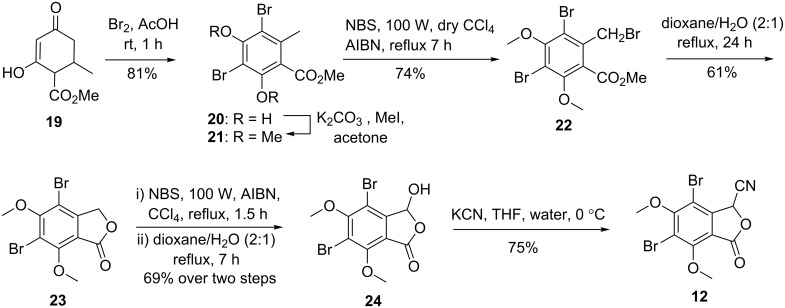
Synthesis of cyanophthalide **12**.

For the synthesis of key synthon **12** (Scheme 2), we started from cyclohexenone **19**, which was prepared by base-catalyzed condensation of methyl acetoacetate with methyl crotonate [24–30]. It was then treated with bromine in AcOH to afford 3,5-dibromoorsellinate **20** in 81% yield [31–33]. Subsequent *O*-methylation of **20** (using CH_3_I, K_2_CO_3_), and benzylic bromination of **21** with NBS followed by lactonization of **22** in a refluxing mixture of dioxane and water afforded phthalide **23** in 61% yield. NBS bromination of **23** afforded the 3-bromophthalide [33], which on treatment with dioxane/water furnished phthalaldehydic acid **24** in 69% yield over two steps [34]. Treatment of **24** with KCN furnished 3-cyanophathalide **12** in 75% yield analogously as described in references [35–37]. The structure of phthalide **12** was confirmed by the appearance of a singlet at δ 5.84 (s, 1H) in the ^1^H NMR spectrum and the appearance of a characteristic band for the C≡N stretching frequency at 2260 cm^−1^ in the IR spectrum. The characteristic carbon for the cyano functionality appeared at δ 111.7 ppm in the ^13^C NMR spectrum.

**Scheme 3:**
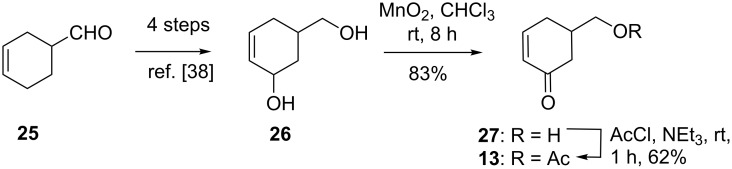
Synthesis of cyclohexenone **13**.

The Michael acceptor **13** was prepared according to the literature procedure starting from cyclohex-3-enecarbaldehyde (**25**) [38]. The diol **26** was oxidized with activated MnO_2_, leading to selective oxidation of the secondary alcohol forming **27** in 83% yield. The cyclohexenone **27** was acetylated with acetyl chloride and pyridine to furnish **13** as an oil in 62% yield (Scheme 3).

**Scheme 4:**
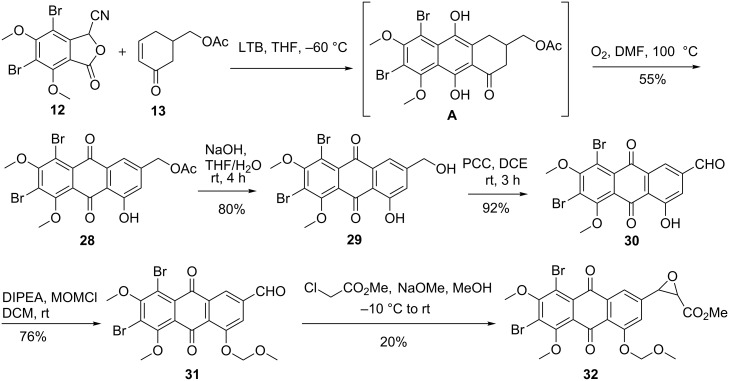
Darzens condensation route to proisocrinins.

In the next stage, Hauser annulation of cyanophthalide **12** with cyclohexenone **13** was carried out in the presence of LiO*t*-Bu (LTB) in THF at −60 °C to furnish quinol **A** [39–42]. Due to its sensitivity to aerial oxidation; it was directly aromatized by bubbling O_2_ through its DMF solution to give anthraquinone **28** in the manner described in [43]. The acetate group in **28** was cleaved with an aqueous alkaline solution to furnish **29** in 80% yield. The alcohol **29** was oxidized to the corresponding aldehyde **30** using PCC in dichloroethane. It was derivatized to its MOM derivative **31** using MOMCl and DIEPA in DCM. Darzens glycidic ester condensation of **31** with methyl 2-chloroacetate and sodium methoxide in methanol (Scheme 4) afforded the desired epoxide **32** [44]. The epoxide **32** was characterized by the signals corresponding to two protons of the epoxide at δ 4.18 and 3.54 [44]. Since the yield of **32** was low, we considered a Horner–Wadsworth–Emmons reaction of aldehyde **31** with triethyl phosphonoacetate as an alternative. Unfortunately, it was not successful, probably due to the interference of the anthraquinone moiety in **31**.

### Second synthetic route

**Scheme 5:**
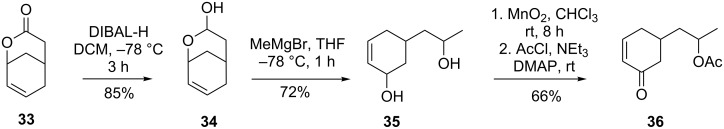
Synthesis of cyclohexenone **36**.

Keeping in view the problems of functionalization of the aldehyde group in **31**, we contemplated the use of already homologated cyclohexenone **36** as the acceptor. Bicyclic lactone **33** [45] was treated with DIBAL-H to afford lactol **34** in 85% yield [46]. Treatment of lactol **34** with methylmagnesium bromide afforded diol **35** in 72% yield. Selective oxidation of the allylic alcohol group in **35** with MnO_2_, followed by acetylation of the secondary hydroxy group with acetyl chloride, triethylamine and DMAP furnished cyclohexenone **36** (Scheme 5).

**Scheme 6:**
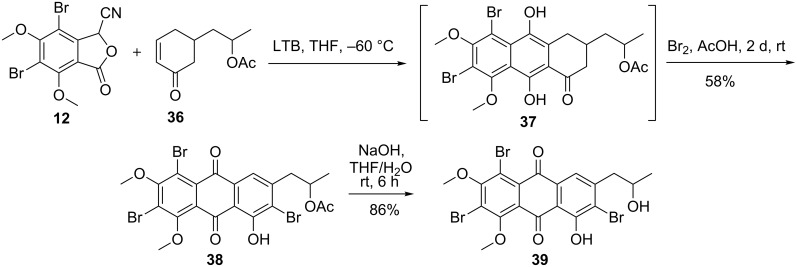
Synthesis of the proisocrinin core structure.

The Hauser annulation of cyanophthalide **12** with acceptor **36** formed hydroquinone **37**, which was directly treated with bromine in DCM to give tribrominated quinone **38** in 58% yield (over two steps) (Scheme 6). The structure of bromo compound **38** was proposed on the basis of the high chemical shift (δ = 7.63 ppm) of the proton attached to the C-4 carbon of the anthraquinone, and its comparison with that in similar structural analogs [47–48]. All attempts to demethylate **38** with BBr_3_ or HBr failed to give the monomethyl analog of **38** [49–52]. The acetate **38** was treated with sodium hydroxide in THF/water (1:1) to give tribromoanthraquinone **39**.

## Conclusion

The Hauser annulation of a dibromophthalide with 5-(2-acetoxypropyl)cyclohexenone has been shown to provide a regiospecific route to the scaffold of proisocrinin F. Further studies on the completion of the synthesis of proisocrinins **6**–**11** are underway.

## Supporting Information

File 1 Detailed experimental procedures, characterization data and copies of ^1^H and ^13^C NMR for all new compounds.
