# Supplementary material for: Regiodefined synthesis of brominated hydroxyanthraquinones related to proisocrinins
Source: Beilstein J Org Chem. 2016 Mar 16;12:531–6. doi: 10.3762/bjoc.12.52 (PMC4901998; doi:10.3762/bjoc.12.52)

Supporting Information

for

Regiodefined synthesis of brominated hydroxyanthraquinones related to proisocrinins

Joyeeta Roy, Tanushree Mal, Supriti Jana and Dipakranjan Mal*

Address: Department of Chemistry, Indian Institute of Technology, Kharagpur-721302, India, Fax: +913222282252

Email: Dipakranjan Mal - dmal@chem.iitkgp.ernet.in

*Corresponding author

Experimental procedures, compound characterization data, and copies of NMR spectra

Table of contents

General remarks..s2

Experimental procedures and compound characterization...............................s2–s10

Copies of spectra...s11–s22

Experimental

General remarks:

All solvents used for chromatography were distilled. Reactions with moisture-sensitive reagents were performed under an inert atmosphere. According to the standard protocols, solvents like DMF, DCM, THF, etc. were dried prior to use. Reactions were monitored by thin-layer chromatography (TLC) carried out on 0.25 mm silica gel plates (60-F254). Products of the reactions were purified by column chromatography on silica gel. Column chromatography was performed over silica gel (60−120 mesh and 230-400 mesh) using hexane and ethyl acetate as eluents. NMR spectra were recorded with a 400 MHz (^{1}H: 400 MHz, ^{13}C: 100 MHz) or a 200 MHz (^{1}H: 200 MHz, ^{13}C: 50 MHz) spectrometer and referenced to the residual solvent peak. ($CHCl_3$, δ_H = 7.26 and δ_C = 77.23; DMSO-d_6, δ_H= 2.50 and δ_C = 39.5). Splitting patterns are indicated as follows: br, broad; s, singlet; d, doublet; dd, double doublet; t, triplet; q, quartet; m, multiplet. Infrared spectra were recorded with FT-IR spectrophotometers and reported in cm^{-1}. Melting points are uncorrected. High-resolution mass spectra were recorded with a mass spectrometer in positive ion mode. The phrase “usual work-up” refers to washing of an organic phase with water (2 × 1/4 the volume of organic phase) and brine (1 × 1/4 the volume of organic phase) and drying (Na_2SO_4), filtration, and concentration under reduced pressure. All known compounds are characterized by comparison of their ^{1}H and ^{13}C NMR spectral data with those reported in the literature. Commercially available starting materials were used without any purification.

4,6-Dibromo-5,7-dimethoxyisobenzofuran-1(3*H*)-one (**23**): To a stirred solution of compound **21** (840 mg, 2.30 mmol) in dry CCl_4 (25 mL), NBS (480 mg, 2.70 mmol)

and a catalytic amount of AIBN were added. The mixture was heated at reflux under a 100 W lamp for 7 h. After completion of reaction, the contents were filtered and the crude bromo product was washed with dry CCl_4. The filtrate was concentrated under vacuum. The solid **22** was dissolved in dioxane:water (2:1, 21 mL) and heated at reflux for 24 h. After completion of the reaction as monitored by TLC analysis usual work-up was carried out. The crude was purified by column chromatography to afford **23** as a white solid in 45% yield (360 mg, 1.03 mmol) over two steps. R_f = 0.3 in 1:5 EtOAc:hexane; mp 186-188 °C; IR (KBr, cm^{-1}) v_{max} 1758, 1612, 1442, 1245, 1059; ^{1}H NMR (200 MHz, $CDCl_3$): δ 5.11 (s, 2H), 4.16 (s, 3H), 3.98 (s, 3H). ^{13}C NMR (50 MHz, $CDCl_3$): δ 166.7, 160.1, 156.8, 148.6, 115.4, 114.6, 105.6, 69.4 (CH_2), 63.0 (CH_3), 61.1 (CH_3).

4,6-Dibromo-3-hydroxy-5,7-dimethoxyisobenzofuran-1(3*H*)-one (**24**)**:** To a stirred solution of the compound **23** (800 mg, 2.28 mmol) in dry CCl_4 (30 mL), NBS (450 mg, 2.51 mmol) and AIBN (ca. 20 mg) were added and the contents were heated at reflux under the exposure of a 100 W bulb for 1.5 h. The mixture was cooled to 0 °C, filtered and then concentrated under reduced pressure to afford a liquid (670 mg). The crude product was then dissolved in dioxane: water (2:1, 20 mL) and heated at 100 °C for 7 h. After completion of the reaction, the usual work-up was performed and the residue was purified by column chromatography to afford phthalaldehydic acid **24** as a solid in 69% yield (577 mg, 1.57 mmol) over two steps. mp 194-196 °C; IR (KBr, cm^{-1}) v_{max} 1734, 1630, 1478, 1293, 938, 770; ^{1}H NMR (400 MHz, DMSO-d_6): δ 6.46 (s, 1H), 3.99 (s, 3H), 3.88 (s, 3H); ^{13}C NMR (100 MHz, DMSO-d_6) δ 164.4, 160.1, 155.7, 148.7, 116.7, 115.8, 107.8, 97.3 (CH), 62.9 (CH_3), 61.2 (CH_3).

5,7-Dibromo-4,6-dimethoxy-3-oxo-1,3-dihydroisobenzofuran-1-carbonitrile (**12**): To a stirred suspension of the phthalaldehydic acid **24** (600 mg, 1.64 mmol) in water (10 mL) and THF (10 mL), KCN (160.2 mg, 2.46 mmol) was added in portions and the mixture was allowed to stir at rt for 10 min, the reaction mixture was then cooled to 0 °C and treated with conc. HCl (6 mL). Stirring was continued again at rt for 1 h and the kept overnight at 0 °C. The reaction mixture was worked up in the usual manner. The crude was purified by column chromatography to afford **12** in 75% yield (460 mg, 1.23 mmol). R_f = 0.3 in 1:5 EtOAc:hexane; mp 180-182 °C; IR (KBr, cm^{-1}) ν_{max} 3468, 2260, 1672, 1256, 1053, 772; ^1H NMR (400 MHz, $CDCl_3$): δ 5.84 (s, 1H), 4.19 (s, 3H), 4.02 (s, 3H); ^{13}C NMR (100 MHz, $CDCl_3$): δ 163.3, 161.3, 157.2, 142.3, 117.3, 113.8, 111.7, 106.5, 65.7 (CH), 63.3 (CH_3), 61.3 (CH_3). HRMS (TOF ESI+): m/z calcd for $C_{11}H_8Br_2NO_4$ $[M + H]^+$ 375.8820, found 375.8827.

(6,8-Dibromo-4-hydroxy-5,7-dimethoxy-9,10-dioxo-9,10-dihydroanthracen-2-yl)methyl acetate (**28**): To a stirred solution of lithium *tert*-butoxide (420 mg, 5.28 mmol) in THF (40 mL) at −60 °C under an inert atmosphere was added a solution of phthalide **12** (600 mg, 1.64 mmol) in THF (5 mL). The resulting yellowish solution was stirred at −60 °C for 25 min, after which a solution of cyclohexenone **13** (300 mg, 1.80 mmol) in THF (5 mL) was added. The cooling bath was removed after 1 h and stirring continued for another 8 h. The reaction was then quenched with 10% NH_4Cl (15 mL). The resulting solution was diluted with ethyl acetate (50 mL) and the layers were separated. The aqueous layer was extracted with ethyl acetate (3 × 25 mL). The combined extracts were processed in usual manner. The resulting crude material was dissolved in minimum quantity of DMF (5 mL), heated at 100 °C, while oxygen bubbling was continuously through the solution for 10-12 h. After addition of

25 mL of water, the solution was up in usual manner. The crude product was purified by column chromatography on silica gel to afford **28** as an orange solid (460 mg, 0.90 mmol) in 55% yield. R_f = 0.4 in 1:5 EtOAc:hexane; mp 170-175 °C, IR (KBr, cm^{-1}) v_{max} 3541, 2940, 1731, 1660, 1586, 1209, 921; ^1H NMR (400 MHz, $CDCl_3$): δ 12.4 (s, 1H), 7.66 (s, 1H), 7.24 (s, 1H), 5.17 (s, 2H), 4.01 (s, 6H), 2.18 (s, 3H); ^{13}C NMR (100 MHz, $CDCl_3$): δ 185.6, 181.4, 170.4, 162.0, 161.4, 159.2, 145.6, 133.8, 133.2, 124.8, 123.7, 121.8 (CH), 117.5 (CH), 115.5, 115.3, 64.6 (CH_2), 62.1 (CH_3), 60.9 (CH_3), 20.8 (CH_3).

1,3-Dibromo-5-hydroxy-7-(hydroxymethyl)-2,4-dimethoxyanthracene-9,10-dione (**29**): To a stirred solution of **28** (500 mg, 0.98 mmol) in THF (5 mL), 50% aqueous NaOH solution (5 mL) was added and the solution stirred for 4 h. After completion of the reaction as monitored by TLC analysis, the reaction mixture was quenched with dilute HCl (5 mL). THF was evaporated under reduced pressure and the solution was worked up in usual manner. The crude was triturated with 1:5 (Hexane: EtOAc) to furnish alcohol **29** as a yellow solid in 80% yield (367 mg, 0.78 mmol). R_f = 0.1 in 2:5 EtOAc:hexane; mp 200-205 °C; ^1H NMR (400 MHz, DMSO-d_6): δ 12.09 (s, 1H), 7.47 (s, 1H), 7.17 (s, 1H), 5.49 (t, J = 11.2 Hz, 1H), 4.53 (d, J = 11.2 Hz, 1H), 3.84 (s, 3H), 3.83 (s, 3H). ^{13}C NMR (100 MHz, DMSO-d_6): δ 185.6, 182.3, 161.3, 160.7, 158.9, 153.3, 134.2, 134.0, 125.9, 123.2, 120.5 (CH), 116.7 (CH), 115.5, 114.7, 62.6 (CH_2), 62.3 (CH_3), 61.3 (CH_3). HRMS (TOF ESI+): m/z calcd for $C_{17}H_{13}Br_2O_6$ $[M + H]^+$ 470.9079, found 470.9085.

6,8-Dibromo-4-hydroxy-5,7-dimethoxy-9,10-dioxo-9,10-dihydroanthracene-2-carbaldehyde (**30**): To a stirred solution of alcohol **29** (400 mg, 0.85 mmol) in

dichloroethane, PCC (276 mg, 1.28 mmol) was added and the mixture stirred at rt for 3 h. After completion of the reaction, the contents were filtered through a celite pad. The filtrate was concentrated under reduced pressure and the residue purified by column chromatography to afford **30** a yellow solid in 92% yield (364 mg, 0.78 mmol). R_f = 0.5 in 2:5 EtOAc:hexane; ^{1}H NMR (400 MHz, $CDCl_3$): δ 12.29 (s, 1H), 10.02 (s, 1H), 8.12 (d, *J* = 2.8 Hz, 1H), 7.67 (d, *J* = 2.8 Hz, 1H), 3.96 (s, 6H). ^{13}C NMR (100 MHz, $CDCl_3$): δ 190.2 (CHO), 186.0, 181.1, 162.2, 162.0, 159.6, 141.6, 134.8, 133.2, 124.9, 124.1, 123.6 (CH), 119.5 (CH), 119.1, 115.9, 62.3 (CH_3), 61.1 (CH_3).

6,8-Dibromo-5,7-dimethoxy-4-(methoxymethoxy)-9,10-dioxo-9,10-dihydroanthracene-2-carbaldehyde (**31**): To a stirred solution of **30** (360 mg, 0.78 mmol) in DCM, DIPEA (0.16 ml, 0.936 mmol) and MOMCl (0.09 mL, 1.17 mmol) were added. After stirring for 3 h at rt, the reaction mixture was diluted with water (10 mL) and extracted with DCM (3 × 40 mL). The combined extracts were subjected to usual work-up. The resulting solution was concentrated and the residue purified by column chromatography to afford **31** (300 mg, 0.58 mmol) as an orange solid in 76% yield. R_f = 0.7 in 2:5 EtOAc:hexane; ^{1}H NMR (400 MHz, $CDCl_3$): δ 10.09 (s, 1H), 8.24 (s, 1H), 7.97 (s, 1H), 5.42 (s, 2H), 4.08 (s, 3H), 3.98 (s, 3H), 3.55 (s, 3H). ^{13}C NMR (100 MHz, $CDCl_3$): δ 190.0 (CH), 181.6, 179.9, 159.9, 157.3, 156.4, 139.7, 136.6, 133.8, 132.0, 128.8, 128.4, 127.8, 127.5, 123.0, 121.7 (CH), 119.3 (CH), 113.6, 95.0 (CH_2), 62.8 (CH_3), 60.6 (CH_3), 56.5 (CH_3). HRMS (TOF ESI+): m/z calcd for $C_{19}H_{15}Br_2O_7$ $[M + H]^+$ 512.9185, found 512.9193.

Methyl 3-(6,8-dibromo-5,7-dimethoxy-4-(methoxymethoxy)-9,10-dioxo-9,10-dihydroanthracen-2-yl)oxirane-2-carboxylate (**32**): To a stirred solution of NaOMe (prepared in situ from 21 mg of Na dissolved in 2 mL of absolute MeOH at -10 °C), 350 mg (0.60 mmol) of aldehyde **31** in 0.1 mL (0.9 mmol) of methyl chloroacetate was slowly added.[11] After 2 h at −5 °C and then for another 4 h at room temperature, the reaction mixture was poured into 5 mL of ice-water containing 0.4 mL of AcOH. The oil which settled at the bottom of beaker was extracted with ethyl acetate (3 x 30 mL). The organic extracts were worked up in usual manner and the residue was flash chromatographed on silica with EtOAc:hexane as the eluents to afford **32** as a light yellow solid in 20% yield (70 mg, 0.12 mmol). R_f = 0.4 in 1:3 EtOAc:hexane; ^{1}H NMR (400 MHz, $CDCl_3$): δ 7.70 (s, 1H), 7.41 (s, 1H), 5.36 (m, 2H), 4.18 (s, 1H), 4.07 (s, 3H), 3.97 (s, 3H), 3.85 (s, 3H), 3.54 (s, 4H) (1H singlet is merged with 3H singlet). ^{13}C NMR (100 MHz, $CDCl_3$): δ 182.7, 180.2, 168.0, 160.1, 157.7, 156.7, 142.2, 136.8, 132.6, 128.2, 124.3, 123.3, 118.3 (CH), 117.6 (CH), 113.9, 95.4 (CH_2), 63.1 (CH), 61.0 (CH), 57.1 (CH_3), 56.9 (CH_3), 56.7 (CH_3), 53.0 (CH). HRMS (TOF ESI+): m/z calcd for $C_{22}H_{19}Br_2O_9$ $[M + H]^+$ 584.9396, found 584.9390.

5-(2-Hydroxypropyl)cyclohex-2-enol (**35**): To a stirred solution of compound **33** (300 mg, 2.17 mmol) in DCM (10 mL) at –78 °C, DIBAL-H (462.9 mg, 3.3 mmol) was added and the mixture stirred for 3 h. The reaction was quenched with saturated sodium potassium tartarate (5 mL) and the mixture worked up in usual manner with DCM. R_f = 0.2 in 1:3 EtOAc:hexane. The product **34** (238.3 mg, 1.7 mmol) was subjected to the next reaction without further purification. It was dissolved in THF (20 mL) and treated with methyl magnesium bromide (1.2 mL of 1.4 M solution, 1.7 mmol) at –78 °C. After 1 h, the reaction was quenched with saturated ammonium

chloride solution (20 mL) and the mixture worked up in usual manner. The crude product was purified by column chromatography (however the diastereomers were not separated) to afford compound **35** as an oil (243 mg, 1.56 mmol) in 72% yield. R_f = 0.2 in 1:5 EtOAc:hexane. The spectral data are reported for a mixture of diastereomers. ^{1}H NMR (400 MHz, $CDCl_3$): δ 5.69 (s, 1H), 5.65-5.62 (m, 1H), 4.28 (s, 1H), 3.94-3.86 (m, 1H), 2.64 (s, 1H), 2.16-2.11 (m, 1H), 2.05-2.01 (m, 1H), 1.82 (s, 1H), 1.70-1.59 (m, 1H), 1.52-1.47 (m, 1H), 1.37-1.30 (m, 1H), 1.24 (t, J = 4.0 Hz, 1H), 1.17 (d, J = 6.0 Hz, 3H). ^{13}C NMR (100 MHz, $CDCl_3$): δ 131.3 (CH), 128.2 (CH), 128.0 (CH), 67.5, 65.1, 64.9, 46.1 (CH_2), 45.1 (CH_2), 39.3 (CH_2), 38.6 (CH_2), 32.2 (CH_2), 31.7 (CH_2), 29.7, 24.1 (CH_3), 23.8 (CH_3). HRMS (TOF ESI+): m/z calcd for $C_9H_{16}NaO_2$ $[M + Na]^+$ 179.1048, found 179.1055.

1-(5-Oxocyclohex-3-enyl)propan-2-yl acetate (**36**): To a stirred solution of diol **35** (2.2 g, 17 mmol) in chloroform (10 mL), activated MnO_2 (14.8 g, 170 mmol) was added and the mixture stirred for 8 h. Another amount (7.4 g, 85 mmol) of MnO_2 was added till completion of the reaction. On completion of the oxidation, the contents were filtered through a sintered funnel. The filtrate was concentrated under reduced pressure and the residue dissolved in DCM (10 mL). This solution was treated with acetyl chloride (2.7 mL, 39.0 mmol), triethyl amine (4.2 mL, 7.8 mmol) and DMAP (10 mg) were sequentially. After 2 h at room temperature, the reaction mixture was quenched with water (10 mL) and worked up in usual manner. The crude product was purified by column chromatography to afford acetate **36** (1 g, 5.14 mmol) as an oil in 66% yield. R_f = 0.4 in 1:5 EtOAc:hexane; ^{1}H NMR 200 MHz, $CDCl_3$): δ 6.95-6.87 (m, 1H), 5.97 (d, J = 10.0 Hz, 1H), 5.03-4.85 (m, 1H), 2.54-2.29 (m, 2H), 2.12-2.06 (m, 2H), 1.97 (s, 1H), 1.71-1.65 (m, 1H), 1.51-1.41 (m, 1H), 1.18 (d, J = 6.2 Hz,

1H). ^{13}C NMR (100 MHz, $CDCl_3$): δ 199.2, 170.6, 149.5, 129.8, 68.4, 44.8, 44.1, 42.0, 41.8 32.3, 31.9, 31.8, 21.4, 20.6, 20.3. HRMS (TOF ESI+): m/z calcd for $C_{11}H_{16}NaO_3$ $[M + Na]^+$ 219.0997, found 219.1001.

1-(3,6,8-Tribromo-4-hydroxy-5,7-dimethoxy-9,10-dioxo-9,10-dihydroanthracen-2-yl)propan-2-yl acetate (**38**): To a stirred solution of lithium *tert*-butoxide (420 mg, 5.28 mmol) in THF (40 mL) at –60 °C (chloroform/liquid N_2 bath) under an inert atmosphere was added a solution of phthalide **12** (600 mg, 1.60 mmol) in THF (5 mL). The resulting yellowish solution was stirred at −60 °C for 25 min, after which a solution of cyclohexenone **36** (352 mg, 1.80 mmol) in THF (5 mL) was added. The resulting mixture was stirred for 8 h at room temperature and then quenched with 10% NH_4Cl (15 mL) and the resulting solution was diluted with ethyl acetate (50 mL). The aqueous layer was extracted with ethyl acetate (3 × 25 mL). The combined extracts were worked in usual manner. The crude product thus obtained was dissolved in AcOH (2 mL) and treated with bromine (0.5 mL, 7.5 mmol) under stirring for 48 h. After completion of the reaction, work-up was done in usual manner. The crude product was purified by column chromatography on silica gel to afford **38** as a brown solid (572 mg, 0.92 mmol) in 58% yield. R_f = 0.35 in 1:5 EtOAc:hexane, mp 170-175 °C, ^1H NMR (400 MHz, $CDCl_3$): δ 13.26 (s, 1H), 7.63 (s, 1H), 5.28 (q, J = 6.4 Hz, 1H), 4.02 (s, 6H), 3.18 (m, 2H), 1.99 (s, 3H), 1.34 (d, J = 6.2 Hz, 3H); ^{13}C NMR (100 MHz, $CDCl_3$): δ 185.4, 181.2, 170.3, 161.7, 159.5, 158.7, 147.4, 133.0, 131.3, 124.6, 123.8, 121.3, 121.2 (CH), 115.6, 114.6, 69.5 (CH), 62.2 (CH_3), 61.0 (CH_3), 43.0 (CH_2), 21.2 (CH_3), 20.1 (CH_3).

1,3,6-Tribromo-5-hydroxy-7-(2-hydroxypropyl)-2,4-dimethoxyanthracene-9,10-dione (**39**): To a stirred solution of **38** (600 mg, 0.95 mmol) in THF (5 mL), 5 mL of 40% aqueous NaOH solution was added and stirred for 6 h. After completion of the reaction as monitored by TLC analysis, the resulting mixture was quenched with dil HCl (5 mL, 6 M). THF was evaporated and the resulting solution was acidified to pH 3 and then worked up was done in usual manner. The crude product was triturated with 1:6 hexane:EtOAc to furnish diol **39** as a yellow solid in 86% yield (482 mg, 0.82 mmol). R_f = 0.3 in 2:5 EtOAc: hexane; ^{1}H NMR (400 MHz, $CDCl_3$): δ 13.24 (s, 1H), 7.66 (s, 1H), 4.25-4.23 (m, 1H), 4.00 (s, 6H), 3.06 (t, *J* = 6.8 Hz, 2H), 1.31 (d, *J* = 6.4 Hz, 3H). ^{13}C NMR (100 MHz, $CDCl_3$): δ 185.7, 181.5, 161.9, 159.7, 158.9, 148.7, 133.3, 131.7, 124.8, 124.0, 121.5, 121.4 (CH), 115.9, 114.7, 67.4 (CH), 62.4 (CH_3), 61.2 (CH_3), 46.6 (CH_2), 23.9 (CH_3). HRMS (TOF ESI+): m/z calcd for $C_{19}H_{16}Br_3O_6$ $[M + H]^+$ 576.8497, found 576.8500.

Copies of spectra

4,6-Dibromo-5,7-dimethoxyisobenzofuran-1(3*H*)-one (**23**)
^{1}H NMR (200 MHz, $CDCl_3$) and ^{13}C NMR (50 MHz, $CDCl_3$)

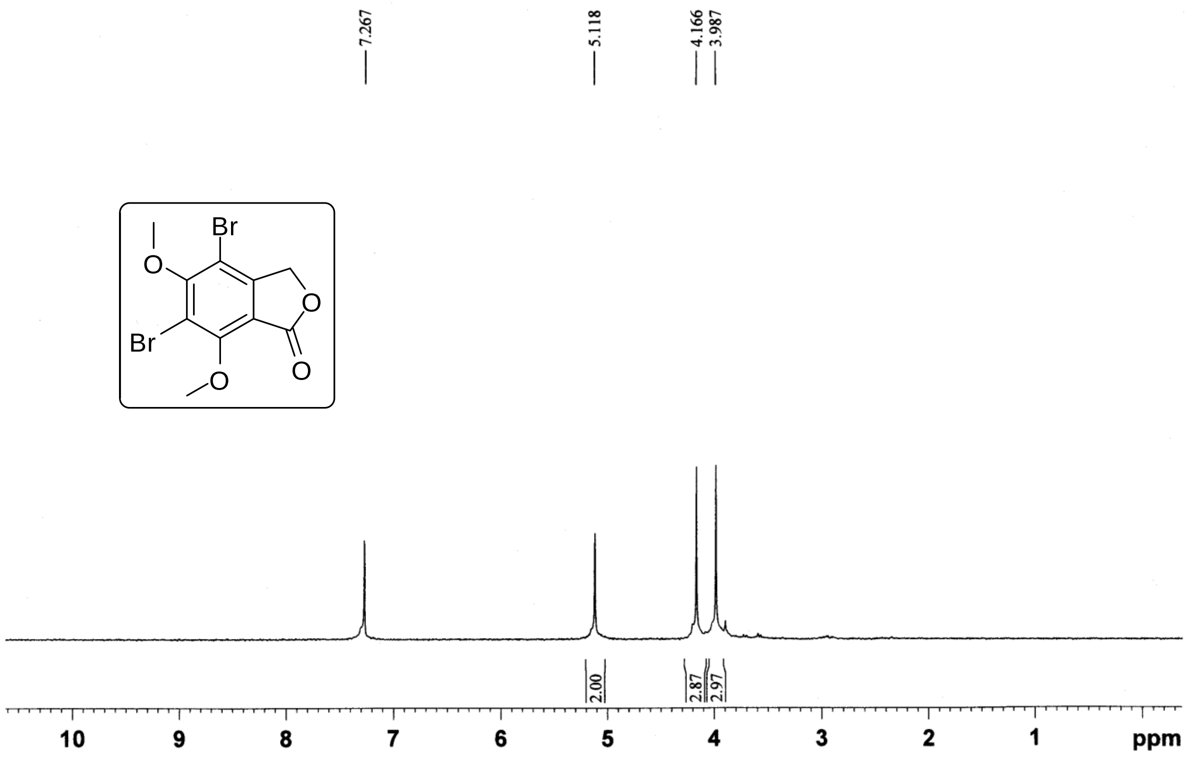

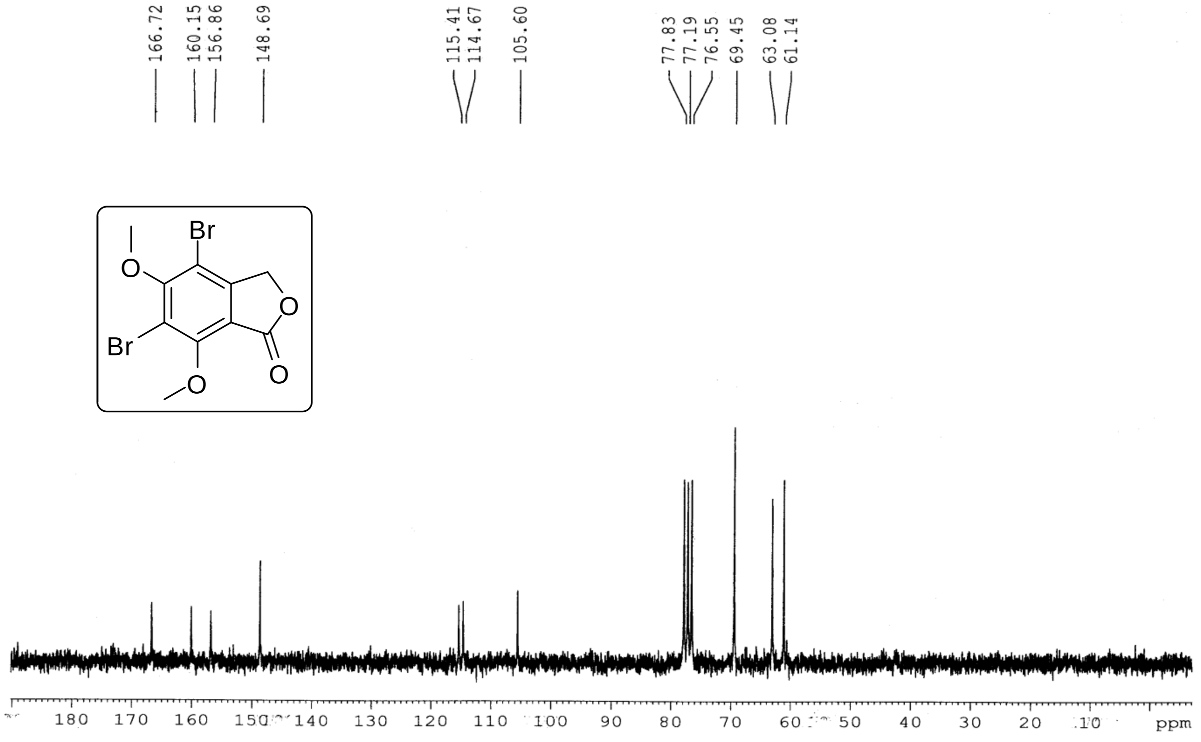

4,6-Dibromo-3-hydroxy-5,7-dimethoxyisobenzofuran-1(3*H*)-one (24)

^{1}H NMR (400 MHz, DMSO-d_6) and ^{13}C NMR (100 MHz, DMSO-d_6)

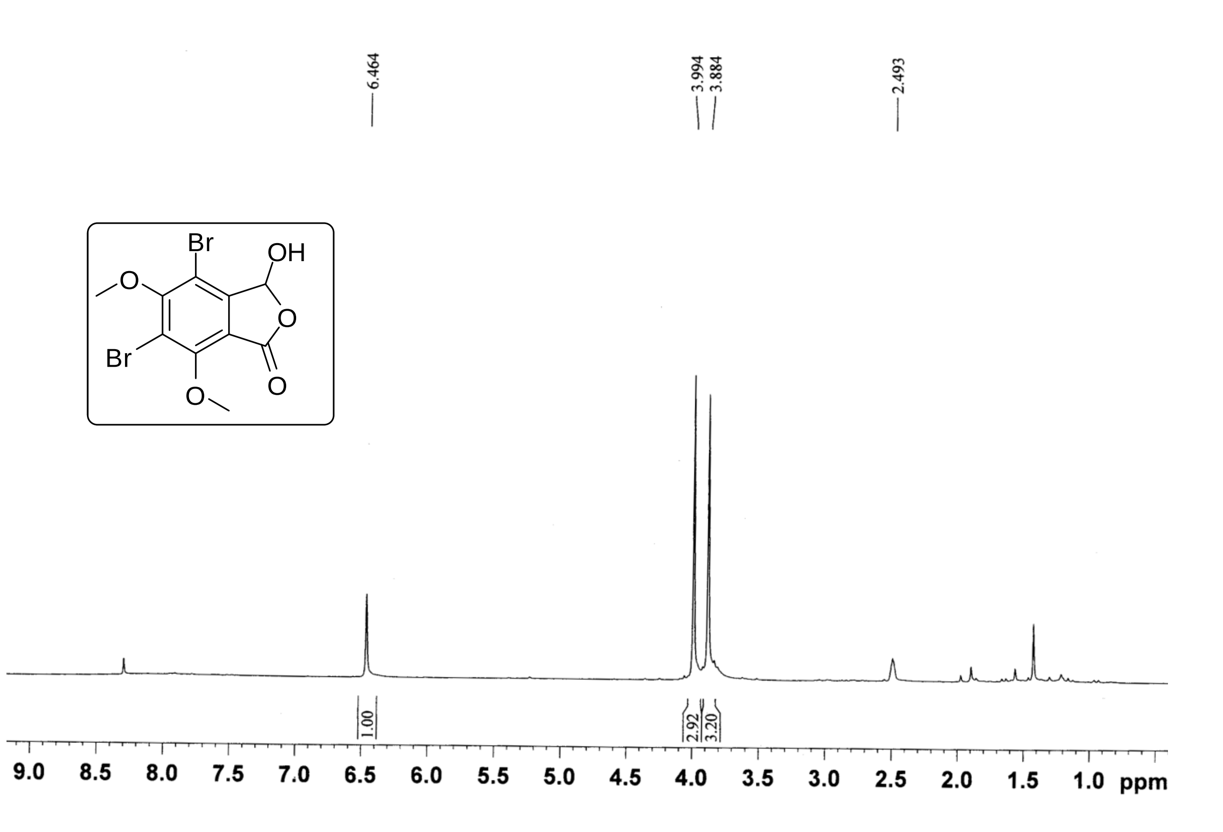

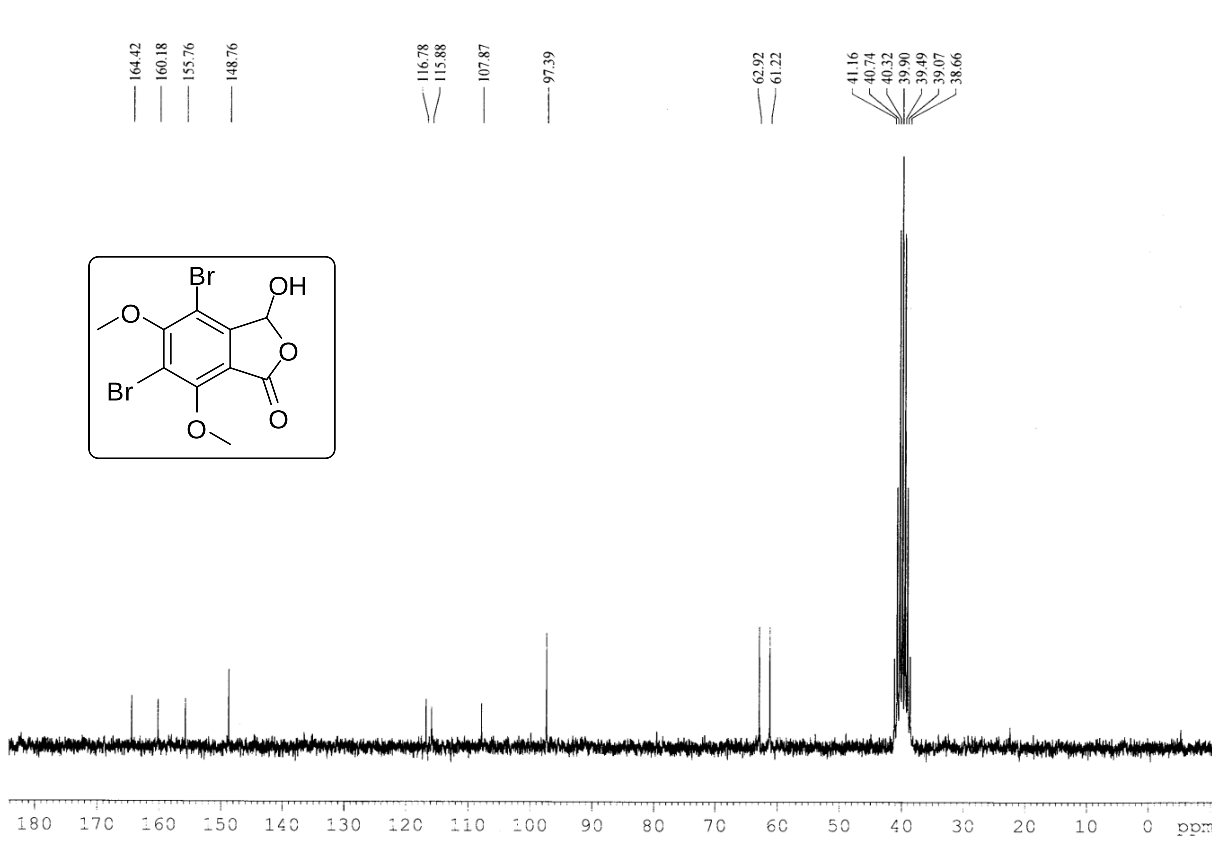

5,7-Dibromo-4,6-dimethoxy-3-oxo-1,3-dihydroisobenzofuran-1-carbonitrile (**12**)

^{1}H NMR (400 MHz, $CDCl_3$) and ^{13}C NMR (100 MHz, $CDCl_3$)

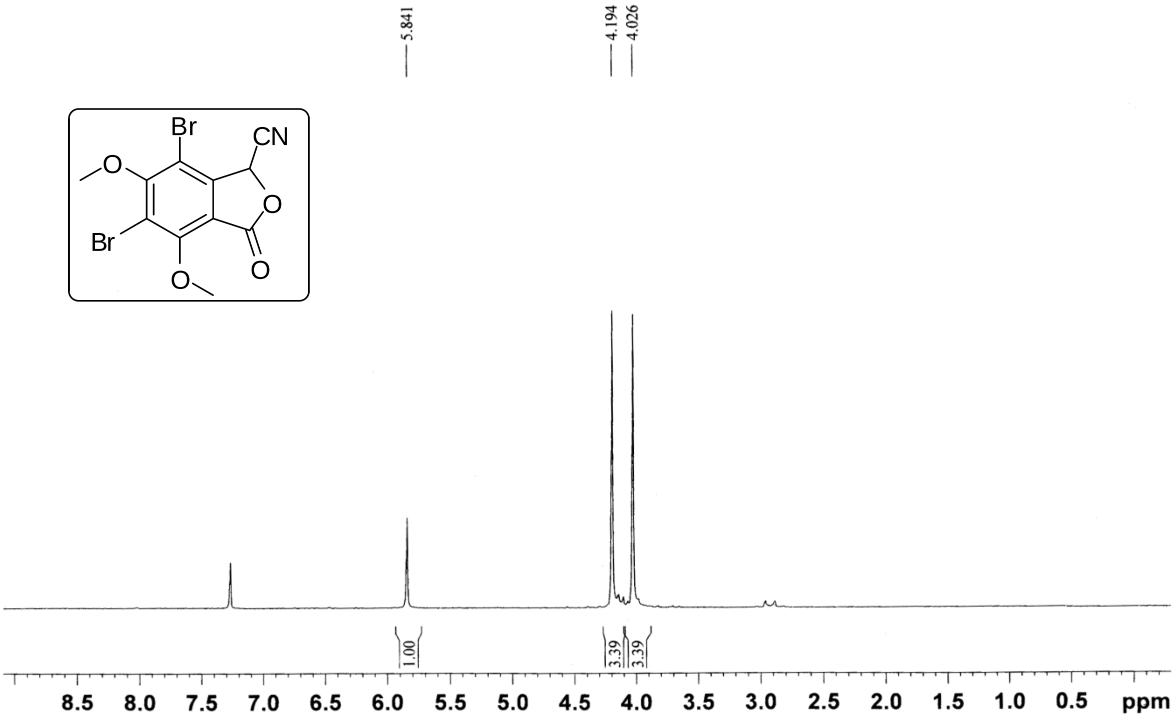

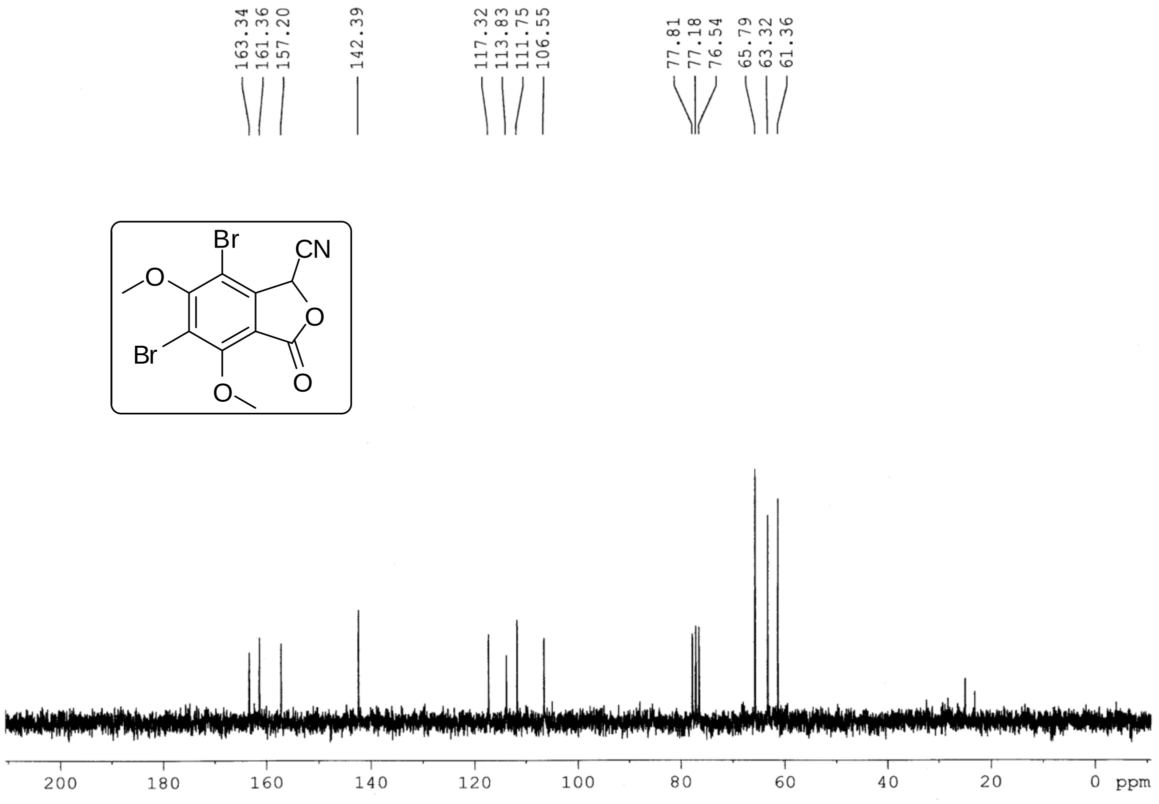

(6,8-Dibromo-4-hydroxy-5,7-dimethoxy-9,10-dioxo-9,10-dihydroanthracen-2-yl)methyl acetate (28)

^{1}H NMR (400 MHz, $CDCl_3$) and ^{13}C NMR (100 MHz, $CDCl_3$)

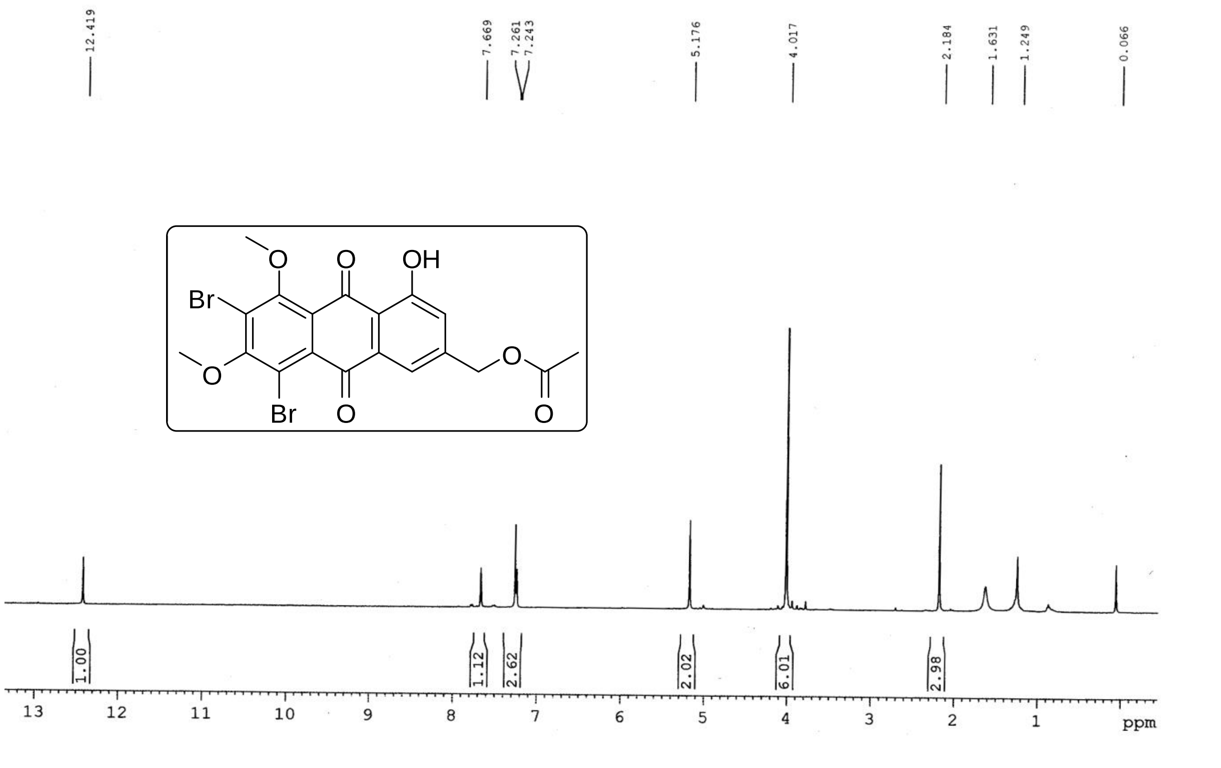

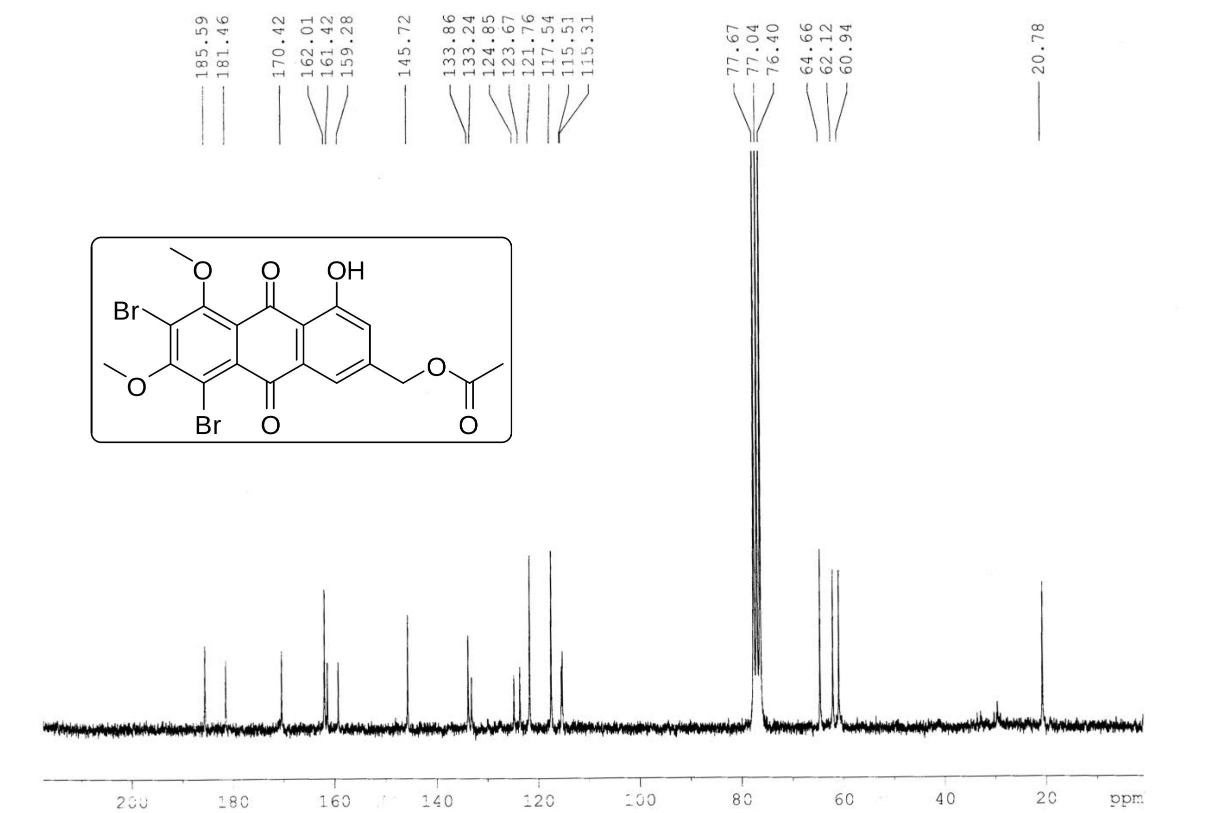

1,3-Dibromo-5-hydroxy-7-(hydroxymethyl)-2,4-dimethoxyanthracene-9,10-dione (**29**)
^{1}H NMR (400 MHz, DMSO-d_6) and ^{13}C NMR (100 MHz, DMSO-d_6)

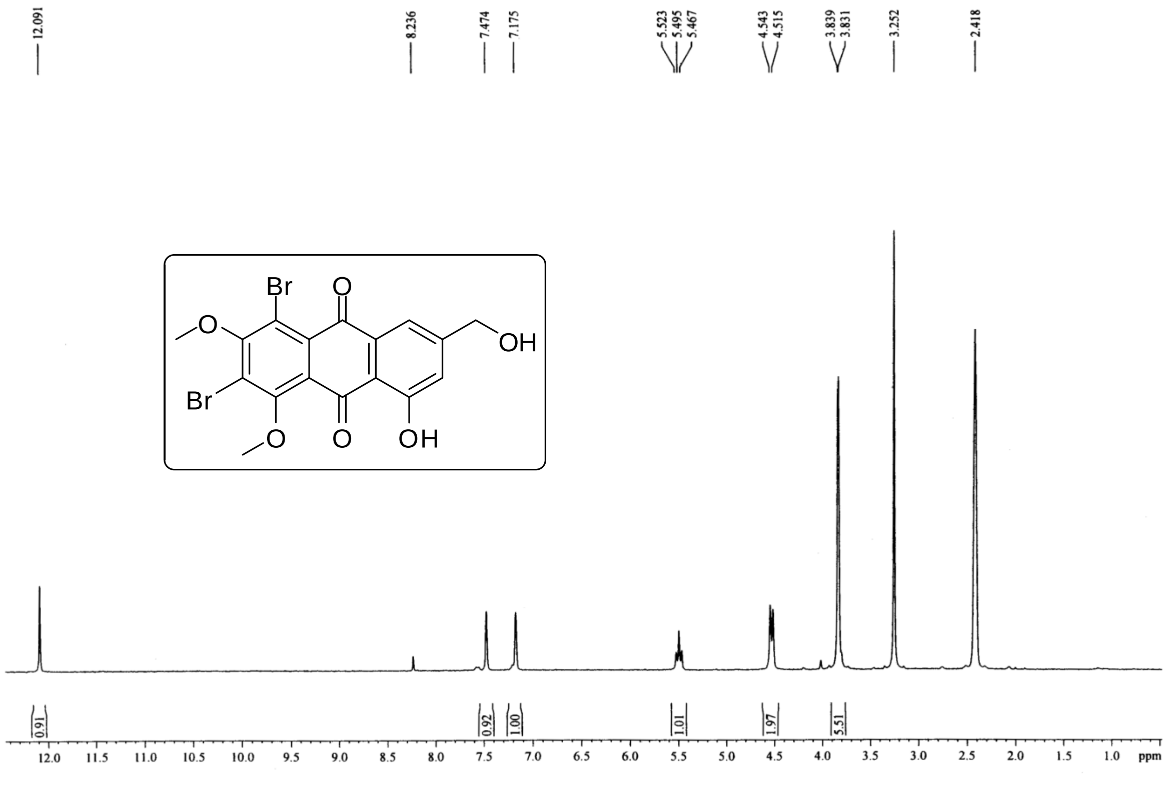

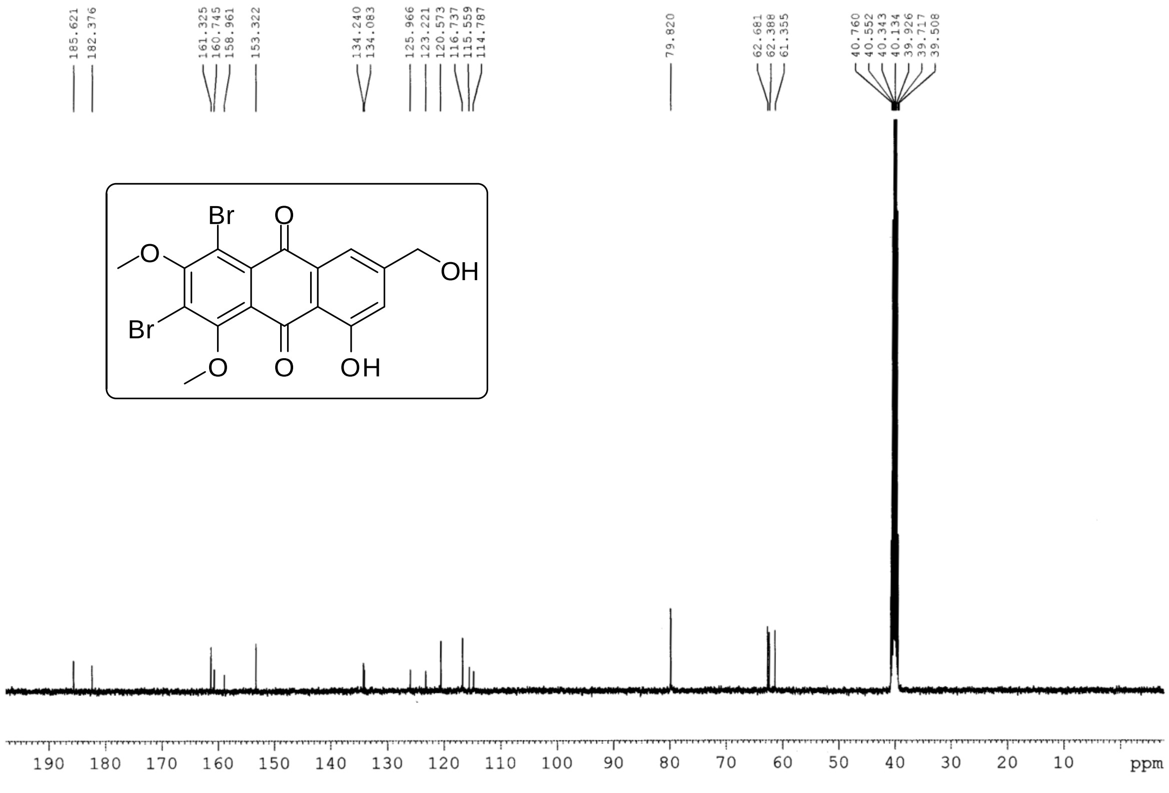

6,8-Dibromo-4-hydroxy-5,7-dimethoxy-9,10-dioxo-9,10-dihydroanthracene-2-carbaldehyde (30)

^{1}H NMR (400 MHz, $CDCl_3$) and ^{13}C NMR (100 MHz, $CDCl_3$)

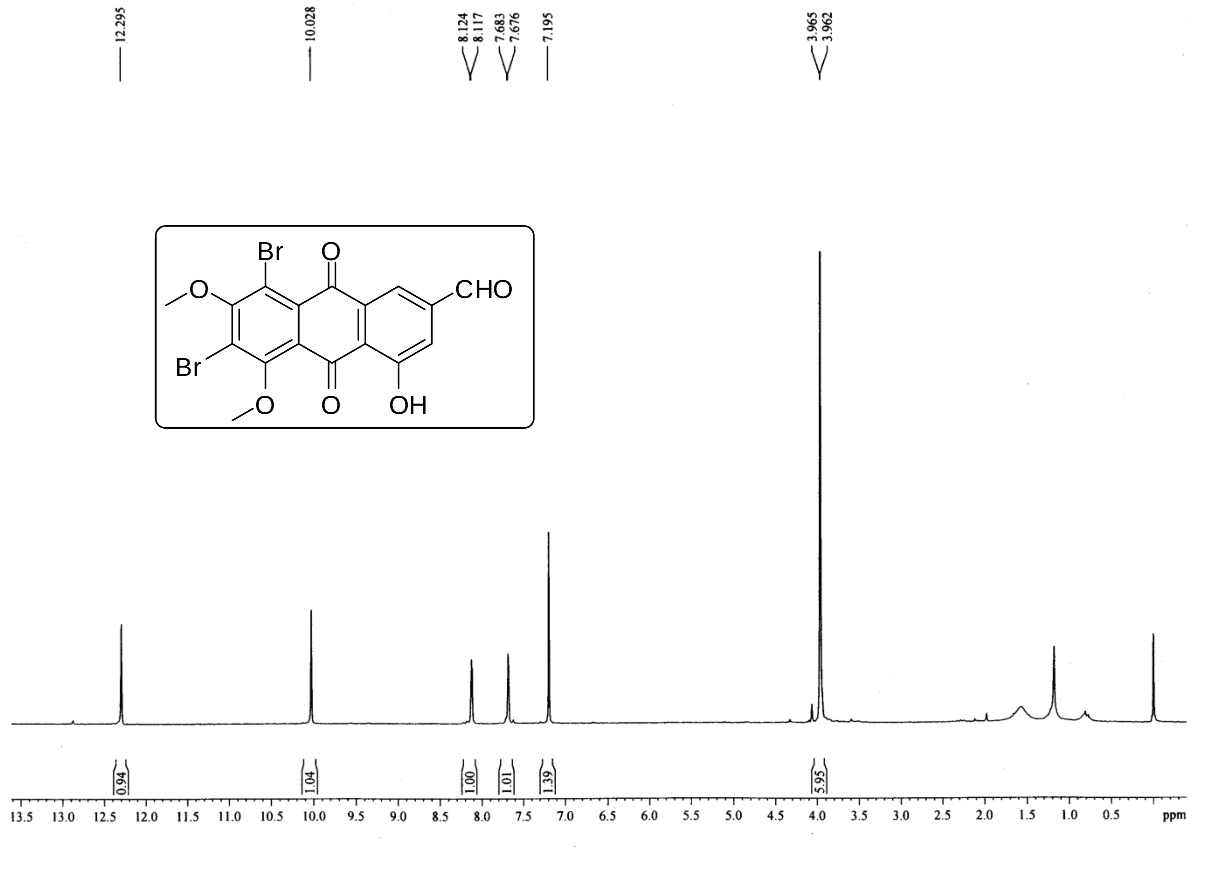

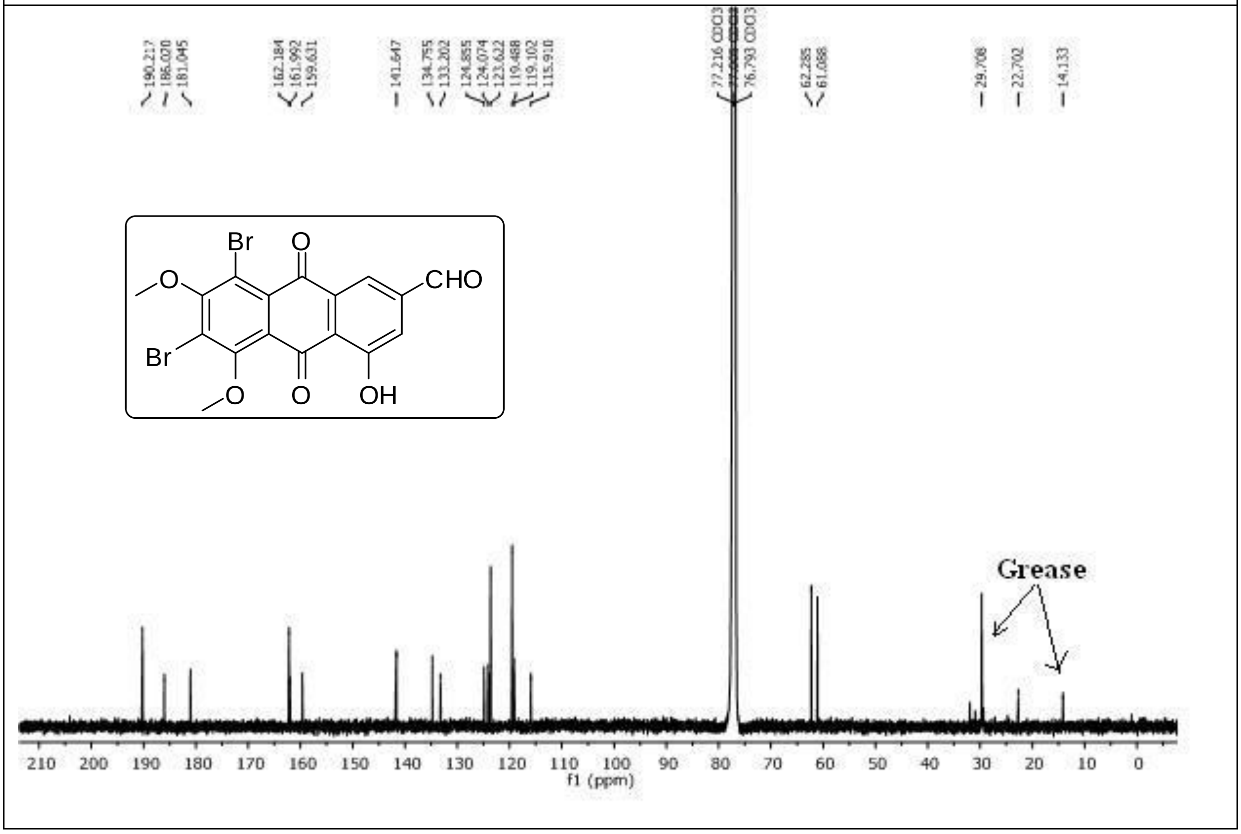

6,8-Dibromo-5,7-dimethoxy-4-(methoxymethoxy)-9,10-dioxo-9,10-dihydroanthracene-2-carbaldehyde (31)

^{1}H NMR (400 MHz, $CDCl_3$) and ^{13}C NMR (100 MHz, $CDCl_3$)

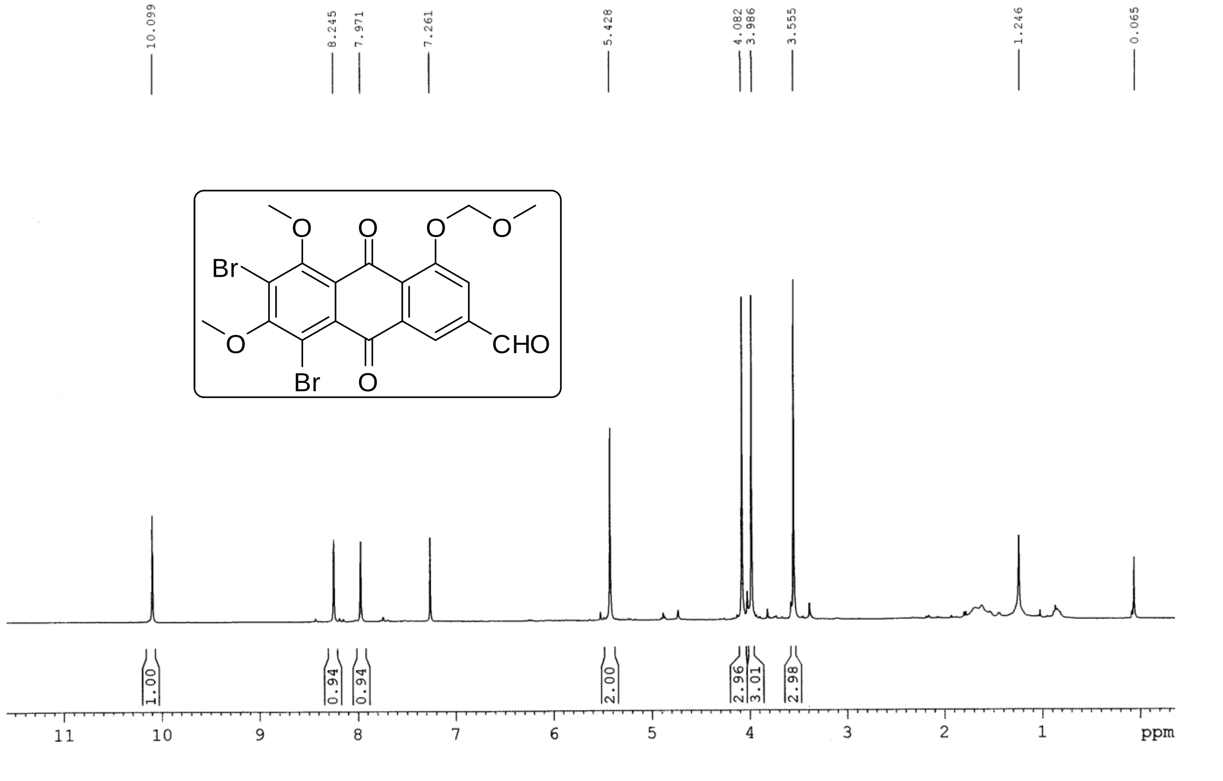

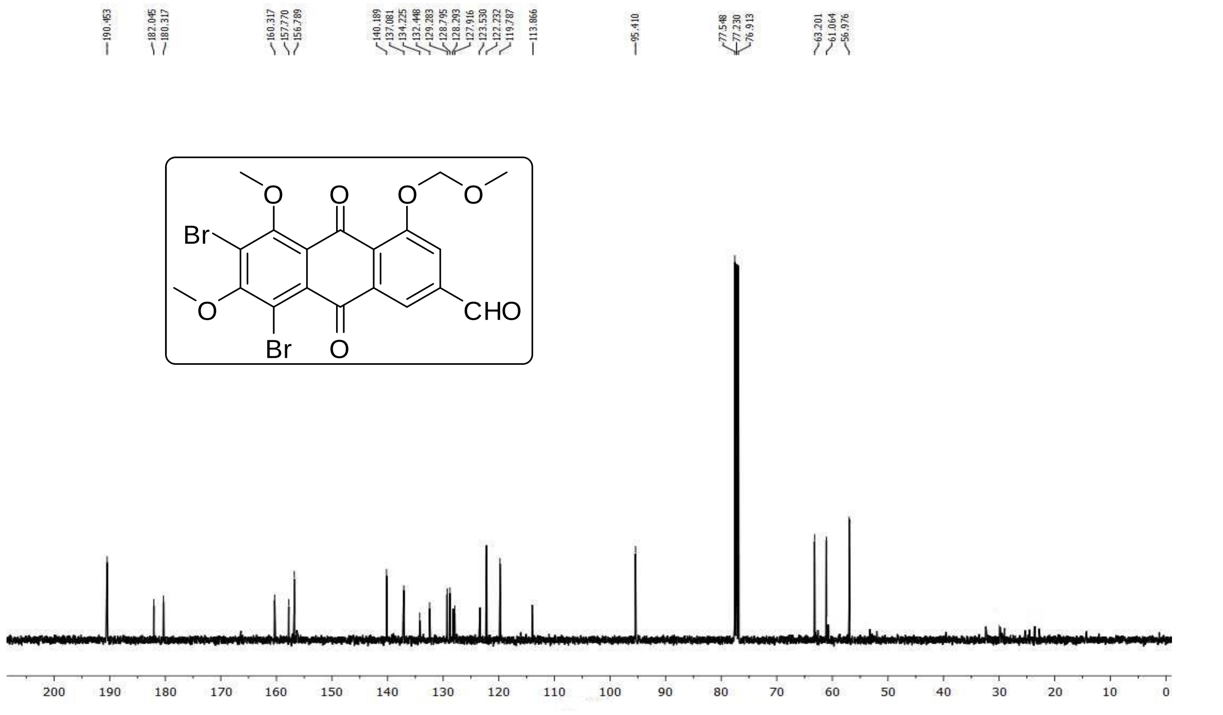

Methyl 3-(6,8-dibromo-5,7-dimethoxy-4-(methoxymethoxy)-9,10-dioxo-9,10-dihydroanthracen-2-yl)oxirane-2-carboxylate (32)

^{1}H NMR (400 MHz, $CDCl_3$) and ^{13}C NMR (100 MHz, $CDCl_3$)

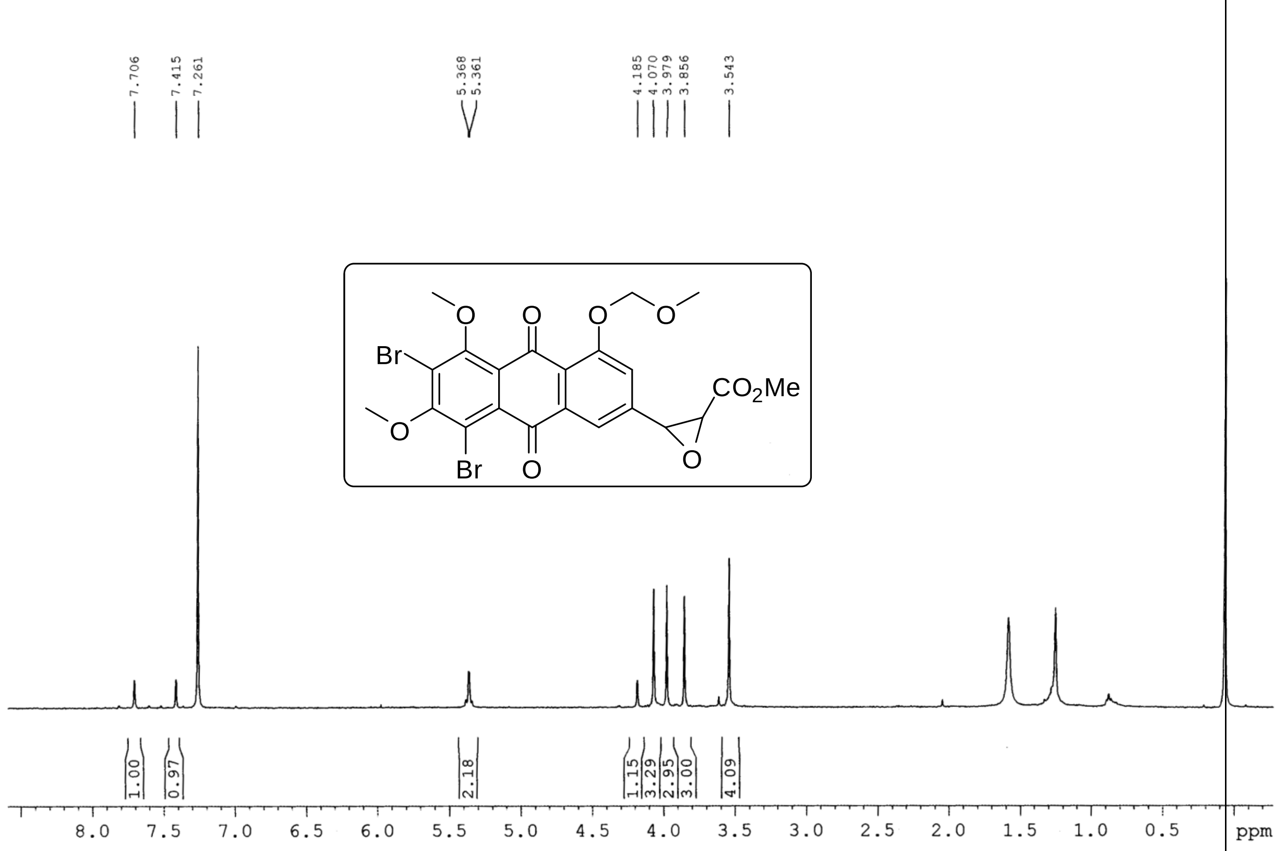

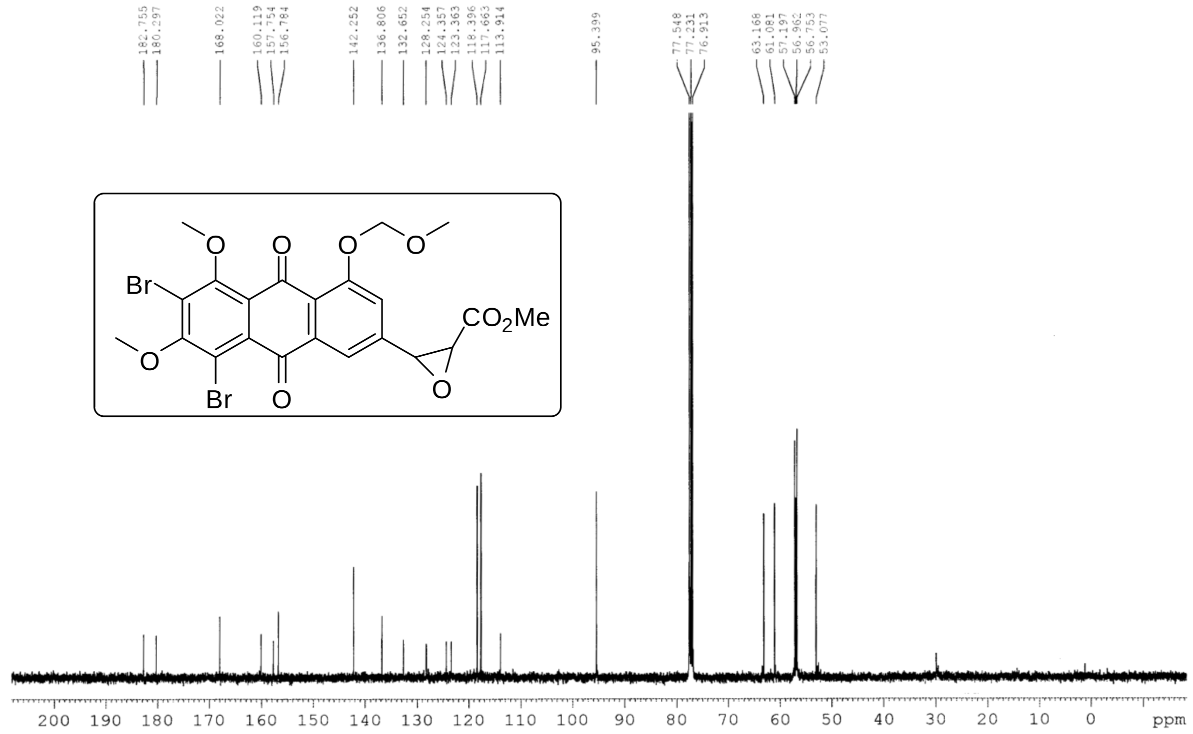

5-(2-hydroxypropyl)cyclohex-2-enol (35)

^{1}H NMR (400 MHz, $CDCl_3$) and ^{13}C NMR (100 MHz, $CDCl_3$)

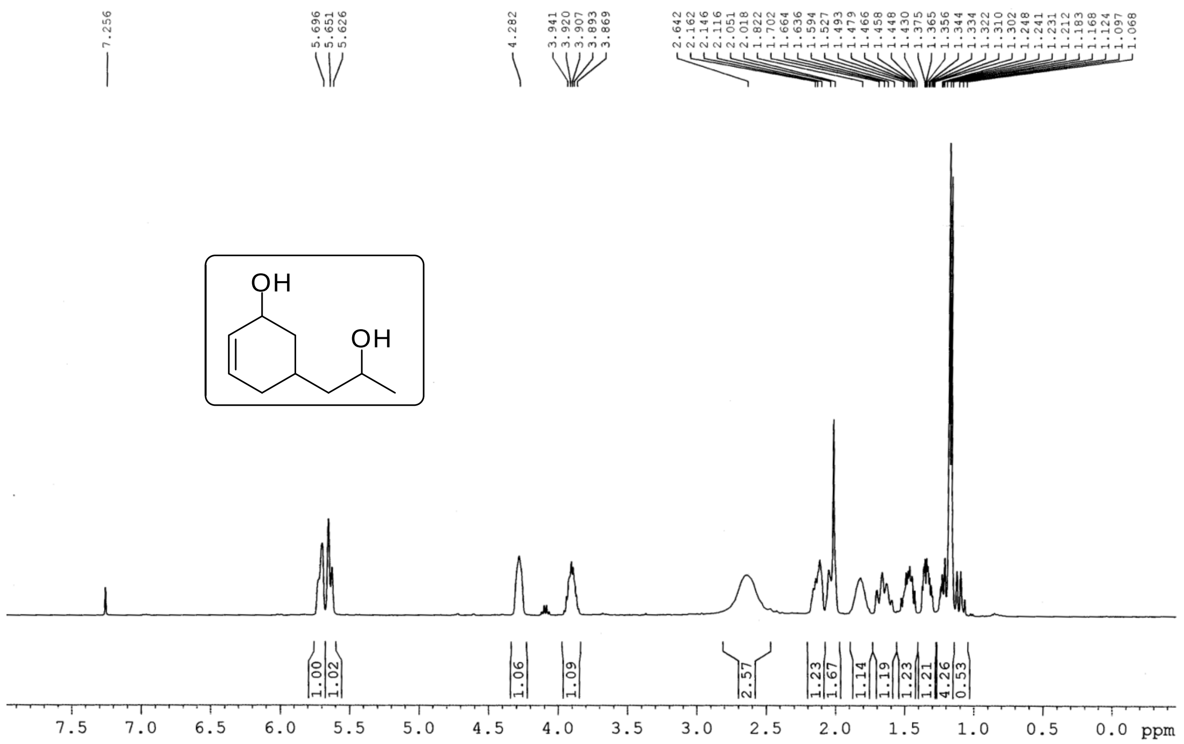

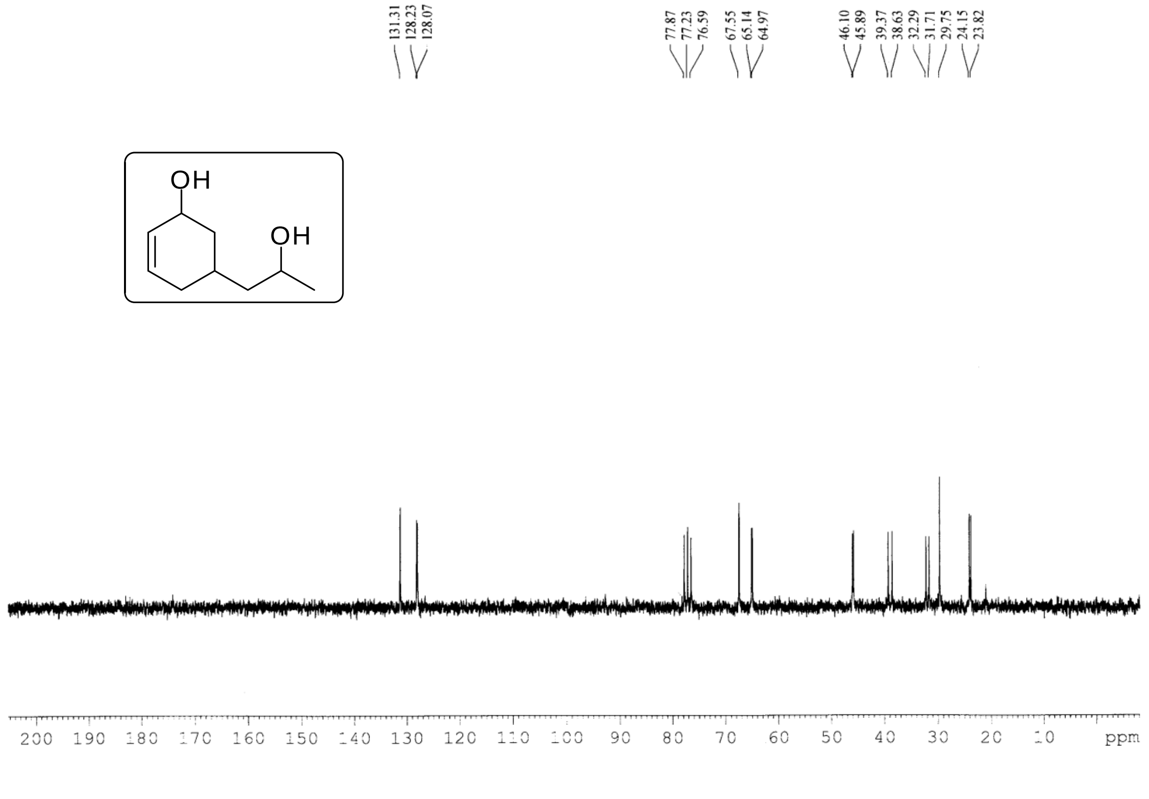

1-(5-oxocyclohex-3-enyl)propan-2-yl acetate (36)

^{1}H NMR (200 MHz, $CDCl_3$) and ^{13}C NMR (100 MHz, $CDCl_3$)

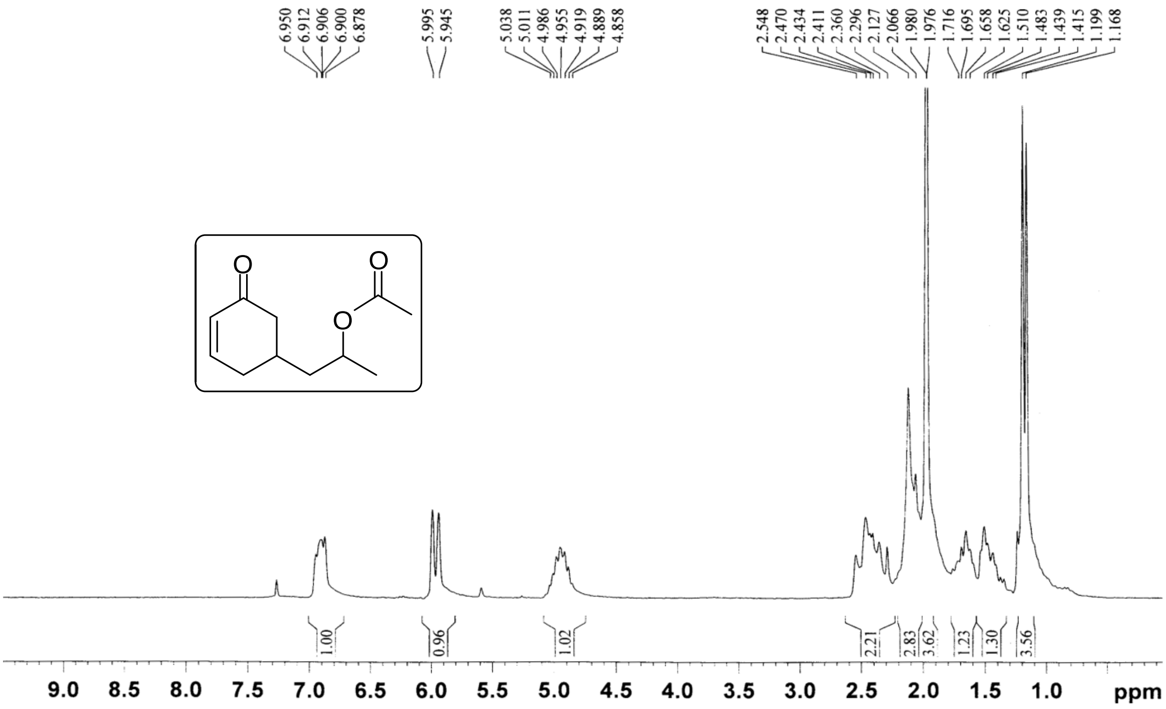

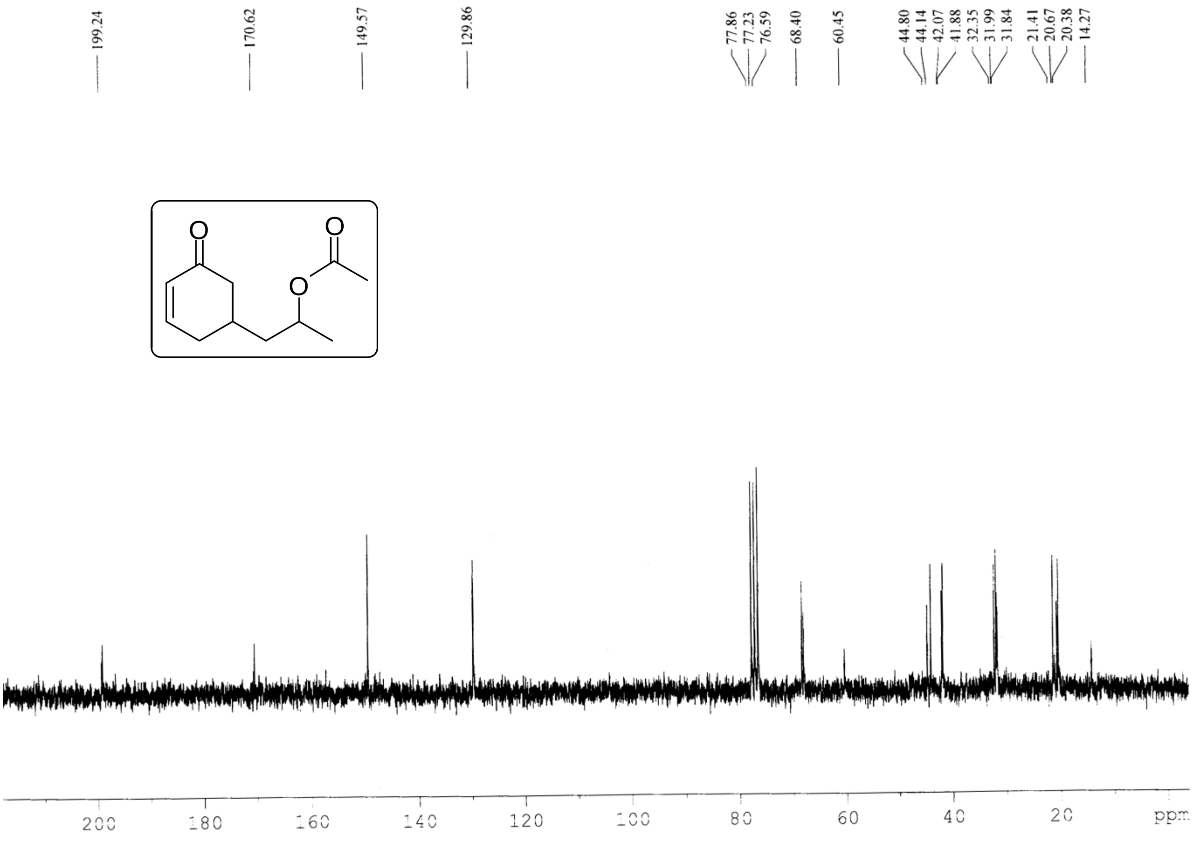

1-(3,6,8-tribromo-4-hydroxy-5,7-dimethoxy-9,10-dioxo-9,10-dihydroanthracen-2-yl)propan-2-yl acetate (**38**)

^{1}H NMR (400 MHz, $CDCl_3$) and ^{13}C NMR (100 MHz, $CDCl_3$)

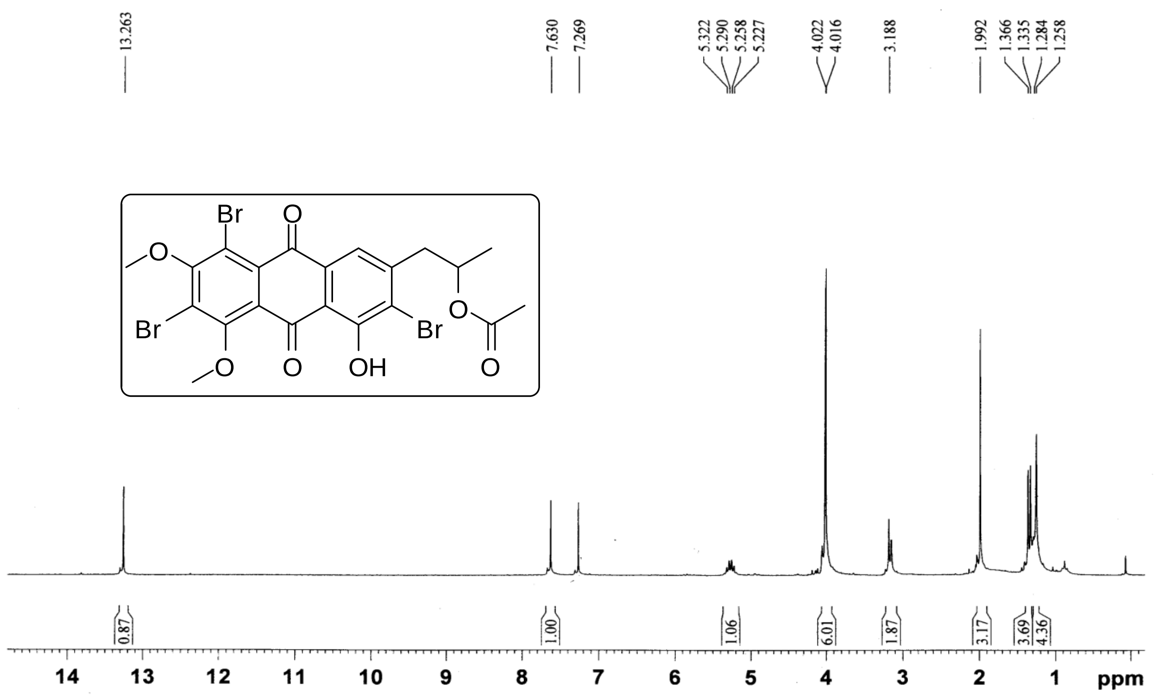

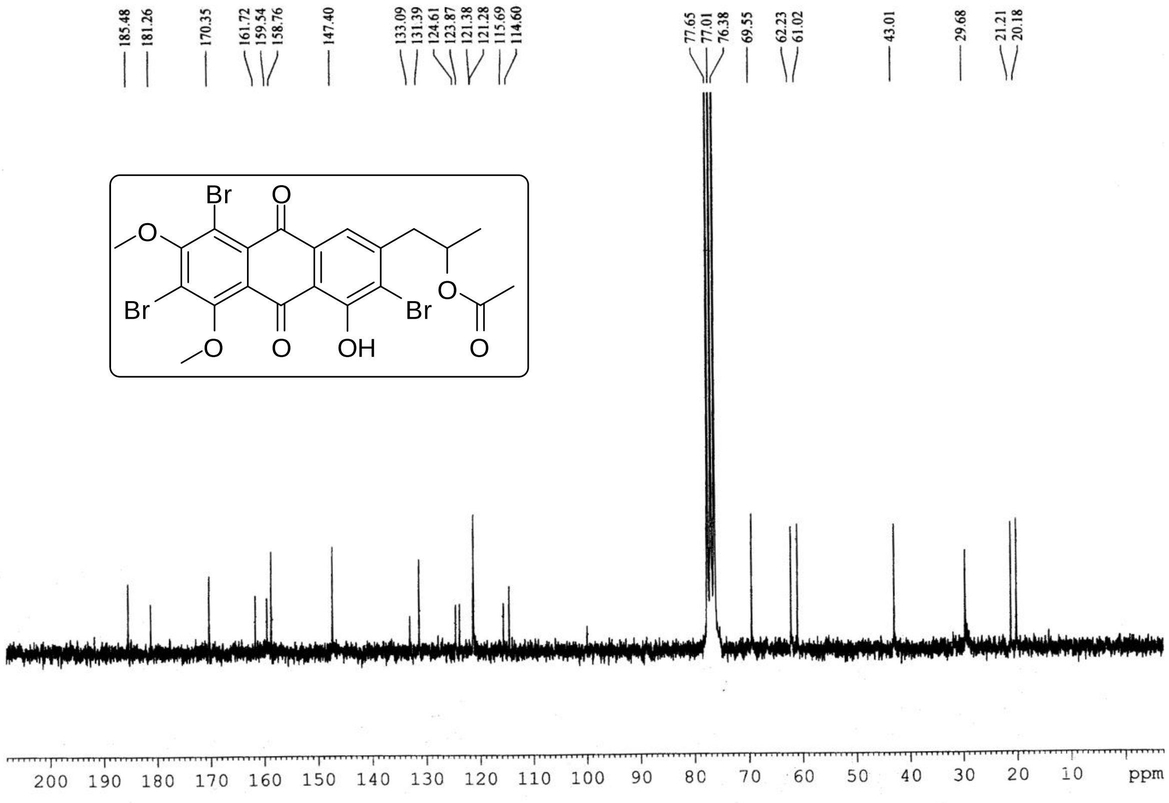

1,3,6-Tribromo-5-hydroxy-7-(2-hydroxypropyl)-2,4-dimethoxyanthracene-9,10-dione (39):

^{1}H NMR (400 MHz, $CDCl_3$) and ^{13}C NMR (100 MHz, $CDCl_3$)

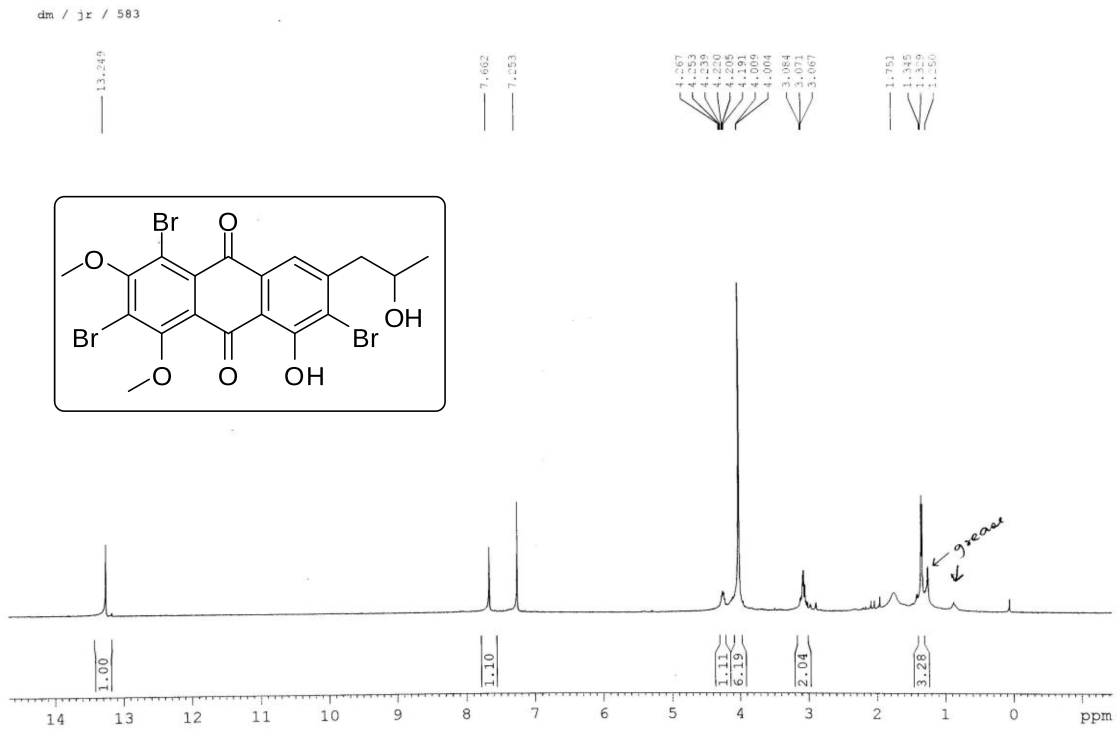

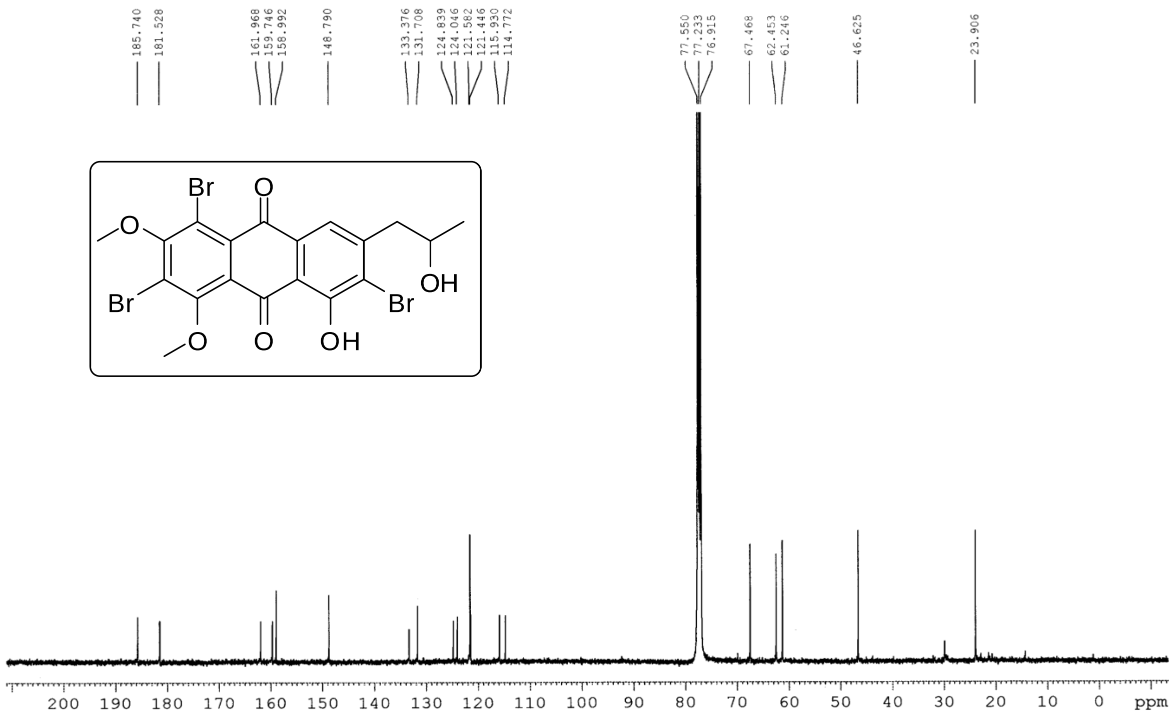

Supplement: File 1 — Detailed experimental procedures, characterization data and copies of 1H and 13C NMR for all new compounds. [file Beilstein_J_Org_Chem-12-531-s001.pdf]
